# Evaluation of Smeared Constitutive Laws for Tensile Concrete to Predict the Cracking of RC Beams under Torsion with Smeared Truss Model

**DOI:** 10.3390/ma14051260

**Published:** 2021-03-07

**Authors:** Mafalda Teixeira, Luís Bernardo

**Affiliations:** Centre of Materials and Building Technologies (C-MADE), Department of Civil Engineering and Architecture, University of Beira Interior, 6201-001 Covilhã, Portugal; mafalda.m.teixeira@ubi.pt

**Keywords:** RC beams, torsion, generalized softened variable angle truss-model (GSVATM), tensile concrete, smeared constitutive law, cracking torque, cracking twist

## Abstract

In this study, the generalized softened variable angle truss-model (GSVATM) is used to predict the response of reinforced concrete (RC) beams under torsion at the early loading stages, namely the transition from the uncracked to the cracked stage. Being a 3-dimensional smeared truss model, the GSVATM must incorporate smeared constitutive laws for the materials, namely for the tensile concrete. Different smeared constitutive laws for tensile concrete can be found in the literature, which could lead to different predictions for the torsional response of RC beams at the earlier stages. Hence, the GSVATM is used to check several smeared constitutive laws for tensile concrete proposed in previous studies. The studied parameters are the cracking torque and the corresponding twist. The predictions of these parameters from the GSVATM are compared with the experimental results from several reported tests on RC beams under torsion. From the obtained results and the performed comparative analyses, one of the checked smeared constitutive laws for tensile concrete was found to lead to good predictions for the cracking torque of the RC beams regardless of the cross-section type (plain or hollow). Such a result could be useful to help with choosing the best constitutive laws to be incorporated into the smeared truss models to predict the response of RC beams under torsion.

## 1. Introduction

In the second half of the last century, the Space Truss Analogy (STA) was successively refined in order to better predict the response of structural concrete beams under torsion. Nowadays, modern truss-based models can be considered reliable, comprehensive and unified analytical models. They are able to simulate the complex 3-dimensional features of the torsional phenomenon, including the nonlinear behavior and the interaction between the material components of the beam in all loading stages. Models based on the STA constitute the basis models for most codes of practice to establish the design procedures for torsion and still continue to be improved and extended [1,2].

A STA-based model assumes that a reinforced concrete (RC) beam under torsion behaves like a cracked thin tube, where the external torque is resisted through a transversal circulatory shear flow. The tube is modeled with a spatial truss, which includes longitudinal and transverse steel reinforcement under tension interacting with inclined concrete struts under compression. The model satisfies the three Navier’s principles of the mechanics of materials, namely, stress equilibrium, strain compatibility and constitutive laws.

Among the STA-based models that have been developed, one of the most commonly used and extended is the Variable-Angle Truss Model (VATM), which was originally proposed by Hsu and Mo in 1985 [3]. This model incorporated for the first time smeared constitutive laws, or smeared stress (σ)—strain (ε) relationships, for both tensile steel reinforcement embedded in concrete and compressive concrete. Such constitutive laws are established from controlled experimental tests on RC panels under in-plane shear, in order to account for, on average (considering an area sufficiently wide to include several cracks), the effect of the biaxial stress state in the principal direction of stresses, the effect of cracking, the interaction between the material components, and both the softening and stiffening effects. The Universal Panel Tester at the University of Houston is one of the testing devices which has most contributed to the establishment of smeared constitutive laws for smeared truss models [4].

Despite being a nonlinear model with an incremental and iterative calculation procedure, the VATM is relatively simple to implement, having access to programming languages in a computer. The model allows us to calculate the full response of RC beams under torsion, namely the torque ($M_{T}$)–twist (θ) curve. The predictions from the VATM showed good agreement with experimental results, namely when predicting the response of RC beams under torsion at the ultimate stage [3,5,6]. When compared with more complex models also proposed for the RC beam under torsion, which sometimes involve large computational effort (for instance [7,8,9,10]), the VATM is recognized as a simpler and more reliable model for predicting the torsional strength of RC beams under torsion, which is one of the most important key parameters for design. It should also be mentioned that smeared approaches, such as the VATM, constitute an alternative approach to local ones in which the local fracture properties are directly accounted for, such as in the numerical models from [11,12]. In smeared approaches, smeared constitutive laws for the materials are incorporated into the model. Such models have been shown to be reliable, on average, for modeling the global behavior of structural elements, such as for the RC beams under torsion.

The VATM has been extended for prestress concrete (PC) beams [13] and also for axially restrained RC beams [14,15]. The VATM was also improved in order to reliably predict the response of RC beam under torsion for the low loading stages, namely the transition between the uncracked stage and the cracked stage. This was achieved by incorporating into the model the contribution of the tensile concrete (neglected in the VATM) through an additional smeared σ—ε constitutive law in the perpendicular direction to the concrete struts. The new model, called generalized softened variable angle truss-model (GSVATM), was proposed in 2015 for RC solid beams under torsion [16]. The predictions from the GSVATM showed good agreement with experimental results for all loading stages. The GSVATM was recently extended for PC beams [17], hollow RC beams [18] and RC flanged beams [19]. A unified version of the model was also recently proposed [2].

The predictions from any smeared truss model, such as the VATM or the GSVATM, strongly depend on the smeared σ—ε relationships for the materials. This important aspect was previously demonstrated by Bernardo et al. in 2012 [20] for the prediction of the torsional strength and corresponding twist for the RC beams under torsion. The study aimed to find the most reliable smeared σ—ε relationships for the materials, among the several ones found in the literature, to be incorporated into the VATM to better predict the ultimate response of RC beams under torsion. The best constitutive laws found in [20], for both the concrete in compression and steel reinforcement in tension, were posteriorly incorporated in the GSVATM [16]. Bernardo et al. in 2012 [20] did not include in their study the prediction of the key parameters for the low loading stages because, as referred to before, the predictions from the VATM were shown to be in good agreement with the experimental results only for the ultimate stage. This is mainly because the model assumes that the member has been fully cracked since the beginning of loading, which is not true. 

For design, it is also important to reliably predict the behavior for the low loading levels. The current codes of practice compel us to check the structural members for both the serviceability and ultimate limit states. For the first one, it is important that the cracking torque is known. As previously referred to, the GSVATM is able to predict the full response of the RC beams under torsion, including the transition between the uncracked stage and the cracked stage. The prediction of such a transition zone highly depends on the smeared constitutive law for the tensile concrete. As for the other constitutive laws referred to (for concrete in compression and steel reinforcement in tension), different proposals of smeared constitutive laws for tensile concrete can be found in the literature. To the best of the authors’ knowledge, no previous study was found with the aim of checking such constitutive laws in smeared truss models, in order to evaluate which features allow the model to give the best predictions for the low loading stages. Usually, researchers working with smeared truss models use their own smeared constitutive laws or choose them based on the proposals from other studies.

In this study, the GSVATM is used to check some proposed smeared constitutive laws for tensile concrete found in the literature. The GSVATM was the chosen model because, as previously stated, it is able to predict the full response of the RC beams under torsion for all loading stages. In addition, this model was proposed by the corresponding author [16] and has also been successfully used in previous studies [2,17,18,19,21]. The chosen parameters to be studied are the cracking torque and the corresponding twist. The theoretical predictions of such parameters are compared with the experimental results from several reported tests on RC beams under torsion. Only RC beams with rectangular sections are studied because they constitute the current solution used in practice. In addition, the number of reported experimental results in the literature for such beams is much higher than for other typologies such as PC beams or beams with a flanged cross-section. 

## 2. The Generalized Softened Variable Angle Truss-Model

For the sake of the readers of this article, a brief description of the GSVATM is presented. The GSVATM was initially proposed for RC plain beams under torsion [16]. Recently, the model was extended and unified for RC hollow beams under torsion [18]. Details about the assumptions of the model, the derivation of the equations and the justification of the calculation solution procedure can be found [16,18].

**Figure 1:**
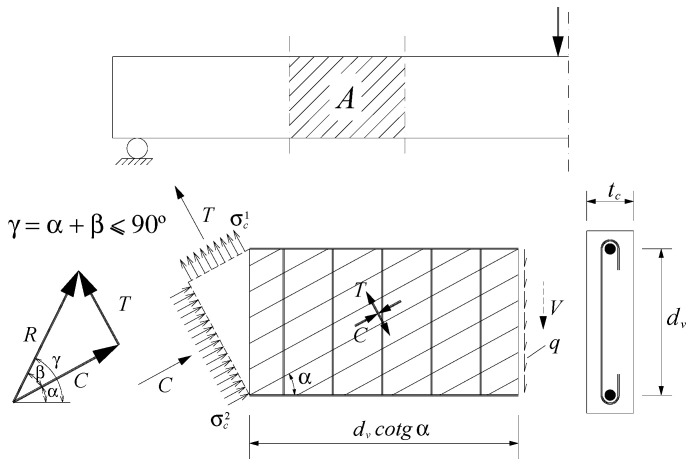
Reinforced concrete (RC) thin beam element [18].

According to the GSVATM, a cracked RC thin beam element under a vertical shear force V, which induces a shear flow q in the cross-section, is modeled with a smeared plain truss analogy, as illustrated in Figure 1. The behavior of the RC thin beam is governed by Equations (1) to (5). The smeared plain truss incorporates inclined concrete struts (with compressive force C) with an angle α to the longitudinal axis, and perpendicular concrete ties (with tensile force T). The corresponding stress fields are denoted by $\sigma_{2}^{c}$ and $\sigma_{1}^{c}$, respectively. The meanings of the parameters are (see Figure 1): R is the resultant force, β is the angle of R to the force C, γ is the angle of R to the longitudinal axis, $t_{c}$ is the width of the cross-section and $d_{v}$ is the distance between centers of the longitudinal bars.

(1) $$R=\sqrt{C^{2}+T^{2}}$$

(2) β=arctan(T/C)

(3) γ=α+β

(4) $$C=\sigma_{2}^{c}t_{c}d_{v}\cos\alpha$$

(5) $$T=\sigma_{1}^{c}t_{c}d_{v}\sin\alpha$$

**Figure 2:**
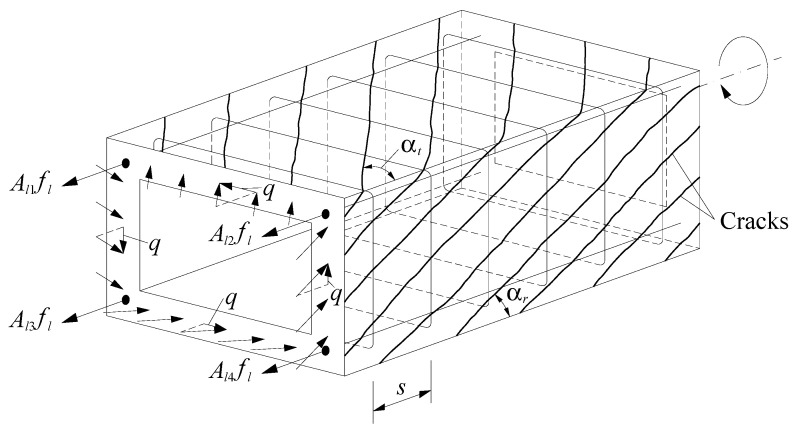
RC hollow beam element [18].

**Figure 3:**
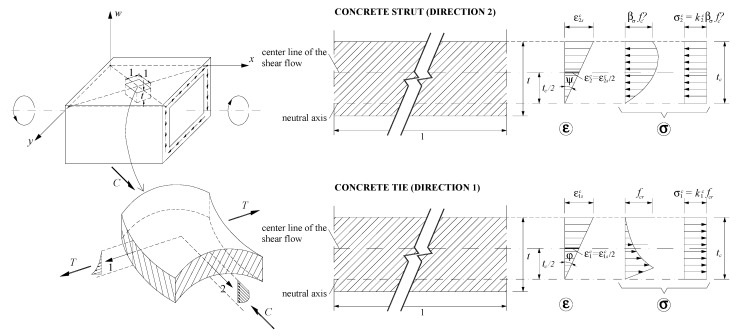
Curvatures and strain gradient in the walls [18].

An equivalent cracked RC hollow beam under a torque $M_{T}$, as illustrated in Figure 2, is modeled as the union of four thin beam elements as in Figure 1. Each thin beam constitutes a wall of the RC hollow beam. As a result of this union, the torque $M_{T}$ induces a circulatory shear flow q and the beam can be modeled with a smeared spatial truss analogy. The center line of the circulatory shear flow q coincides with the center line of the walls. The behavior of the RC hollow beam is governed by equilibrium equations, Equations (6) to (8), and compatibility equations, Equations (9) to (12). If $\gamma =\alpha +\beta >90^{\circ }$, Equation (7) must be multiplied by (−1). The previous equations account for the strain gradient along the walls’ thickness due to the bidirectional opposite curvatures induced by bending (Figure 3).

(6) $$M_{T}=\frac{2A_{ο}R\sin\gamma }{d_{v}}$$

(7) $$t_{c}=\frac{A_{sl}f_{sl}}{\sigma_{2}^{c}p_{ο}}\frac{\cos\beta }{\cos\alpha \cos\gamma }\;\mathrm{for}\;\gamma =\alpha +\beta \leq 90^{\circ }$$

(8) $$\alpha =\arctan\left(\frac{\sqrt{F^{2}{\left(\tan\beta \right)}^{2}+F{\left(\tan\beta \right)}^{4}+F+{\left(\tan\beta \right)}^{2}}}{F{\left(\tan\beta \right)}^{2}+1}\right)\;\mathrm{with}\;F=\frac{A_{st}f_{st}p_{ο}}{A_{sl}f_{sl}s}$$

(9) $$\varepsilon_{\mathrm{st}}=\left(\frac{A_{o}^{2}\sigma_{2}^{c}\sin\gamma }{p_{ο}M_{T}\cos\beta \tan\alpha \sin\alpha }-\frac{1}{2}\right)\varepsilon_{2s}^{c}$$

(10) $$\varepsilon_{\mathrm{sl}}=\left(\frac{A_{o}^{2}\sigma_{2}^{c}\sin\gamma }{p_{ο}M_{T}\cos\beta \cot\alpha \sin\alpha }-\frac{1}{2}\right)\varepsilon_{2s}^{c}$$

(11) $$\theta =\frac{\varepsilon_{2s}^{c}}{2t_{c}\sin\alpha \cos\alpha }$$

(12) $$\varepsilon_{1s}^{c}=2\varepsilon_{1}^{c}=2\varepsilon_{\mathrm{sl}}+2\varepsilon_{\mathrm{st}}+\varepsilon_{2s}^{c}$$

In the previous equations (see Figure 2 and Figure 3), $t_{c}$ is the effective thickness of the concrete strut and tie in the walls, $A_{ο}=\left(x-t_{c}\right)\left(y-t_{c}\right)$ and $p_{ο}=2\left(x-t_{c}\right)+2\left(y-t_{c}\right)$ are the area enclosed and the perimeter of the center line of the shear flow q (with x the minor and y the major outer dimension of the beam’s cross-section), respectively, $A_{sl}$ is the total area of the longitudinal reinforcement, $A_{st}$ is the area of one rebar of the transverse reinforcement, s is the longitudinal spacing of the transverse reinforcement, $f_{sl}$ and $f_{st}$ are the tensile stresses in the longitudinal and transverse reinforcement, respectively, $\varepsilon_{\mathrm{sl}}$ and $\varepsilon_{\mathrm{st}}$ are the tensile strains in the longitudinal and transverse reinforcement, respectively, $\varepsilon_{2s}^{c}$ and $\varepsilon_{1s}^{c}$ are the strains at the outer fiber of the concrete strut and concrete tie, respectively, $\varepsilon_{1}^{c}$ is the average strain in the concrete tie, and θ is the twist per unit length.

As referred to in the introduction section, the GSVATM incorporates smeared σ—ε relationships to model the behavior of the compressive concrete in the struts, the tensile concrete in the ties and the tensile longitudinal and transverse steel reinforcement (rebars embedded in concrete). For the RC beams under torsion, some suitable smeared σ—ε relationships were previously found by Bernardo et al. in 2012 [20] and are also used in this study. For the compressive concrete, the smeared σ—ε relationship proposed by Belarbi and Hsu in 1995 [22] (Equations (13) and (14)) with softening factor $\beta_{*}=\beta_{\sigma }=\beta_{\varepsilon }$, for both the peak stress and corresponding strain, proposed by Zhang and Hsu in 1998 [23] (Equations (15) to (18)) are used. For the steel reinforcement in tension, the smeared σ—ε relationship proposed by Belarbi and Hsu in 1994 [24] (Equations (19) to (21)) is used.

The meaning of the parameters are: $f_{c}^{\prime }$ is the average uniaxial concrete compressive strength, $\varepsilon_{ο}$ is the strain corresponding to $f_{c}^{\prime }$, $\varepsilon_{2}^{c}$ is the average strain in the concrete strut (Figure 3), $\rho_{l}$ and $\rho_{t}$ are the longitudinal ($\rho_{l}=A_{sl}/A_{c}$, with $A_{c}=xy$) and transverse ($\rho_{t}=A_{st}u/A_{c}s$, with u=2x+2y) reinforcement ratios, respectively, $f_{ly}$ and $f_{ty}$ are the yielding stresses for the longitudinal and transverse reinforcement, respectively, $f_{cr}$ is the tensile concrete strength, $\varepsilon_{\mathrm{cr}}$ is the strain corresponding to $f_{cr}$, $f_{s}$ and $\varepsilon_{s}$ are the stress and strain in the steel reinforcement (longitudinal or transversal), respectively, $E_{s}$ is the Young’s Modulus for steel reinforcement, $f_{y}$ is the yielding stress of steel reinforcement (longitudinal or transversal) and ρ is the reinforcement ratio (longitudinal or transversal).

(13) $$\sigma_{2}^{c}=\beta_{\sigma }f_{c}^{\prime }\left[2\left(\frac{\varepsilon_{2}^{c}}{\beta_{\varepsilon }\varepsilon_{ο}}\right)-{\left(\frac{\varepsilon_{2}^{c}}{\beta_{\varepsilon }\varepsilon_{ο}}\right)}^{2}\right]{\;\mathrm{if}\;\varepsilon }_{2}^{c}\leq \beta_{\varepsilon }\varepsilon_{ο}$$

(14) $$\sigma_{2}^{c}=\beta_{\sigma }f_{c}^{\prime }\left[1-{\left(\frac{\varepsilon_{2}^{c}-\beta_{\varepsilon }\varepsilon_{ο}}{2\varepsilon_{ο}-\beta_{\varepsilon }\varepsilon_{ο}}\right)}^{2}\right]{\;\mathrm{if}\;\varepsilon }_{2}^{c}>\beta_{\varepsilon }\varepsilon_{ο}$$

(15) $$\beta_{*}=\beta_{\sigma }=\beta_{\varepsilon }=\frac{R\left(f_{c}^{\prime }\right)}{\sqrt{1+\frac{400\varepsilon_{1}^{c}}{\eta \prime }}}$$

(16) $$\eta =\frac{\rho_{l}f_{ly}}{\rho_{t}f_{ty}\;}$$

(17) $$\{\begin{array}{c} \eta \leq 1\Rightarrow \eta \prime =\eta \\ \eta >1\Rightarrow \eta \prime =1/\eta \end{array}$$

(18) $$R\left(f_{c}^{\prime }\right)=\frac{5.8}{\sqrt{f_{c}^{\prime }(\mathrm{MPa})}}\leq 0.9$$

(19) $$f_{s}=\frac{0.975E_{s}\varepsilon_{s}}{{\left[1+{\left(\frac{1,1E_{s}\varepsilon_{s}}{f_{y}}\right)}^{m}\right]}^{\frac{1}{m}}}+0.025E_{s}\varepsilon_{s}$$

(20) $$m=\frac{1}{9B-0.2}\leq 25$$

(21) $$B=\frac{1}{\rho }{\left(\frac{f_{cr}}{f_{y}}\right)}^{1.5}$$

For the tensile concrete, the smeared σ—ε relationships checked in this study are presented in more detail in the Section 3. However, in order to present the equations for some correction coefficients and also the flowchart with the calculation procedure for the GSVATM, the following general and common form of the equations are written (F(…) stands for “function of …”):

(22) $$\sigma_{1}^{c}=E_{c}\varepsilon_{1}^{c}{\;\mathrm{if}\;\varepsilon }_{1}^{c}\leq \varepsilon_{\mathrm{cr}}$$

(23) $$\sigma_{1}^{c}=F\left(f_{cr};\varepsilon_{1}^{c}\right){\;\mathrm{if}\;\varepsilon }_{1}^{c}>\varepsilon_{\mathrm{cr}}$$

In Equation (22), which models the linear–elastic stage before cracking, $E_{c}$ is the Young’s Modulus for the concrete. Based on the proposals from previous studies [9,25,26], and in order to unify the GSVATM for both the RC plain and the hollow beams under torsion, and also to improve the predictions from the model for the low loading stages, in 2019 Bernardo [18] presented a set of equations (Equations (24) to (29)) to compute the parameters $\varepsilon_{\mathrm{cr}}$ and $E_{c}$, accounting for the correction coefficients μ and λ. These equations apply for all smeared σ—ε relationships for tensile concrete presented in the Section 3 and checked in this study.

(24) $$\varepsilon_{\mathrm{cr}}=0.00008\mu$$

(25) $$E_{c}=3875\lambda \sqrt{f_{c}^{\prime }\;(\mathrm{MPa})}$$

(26) μ=λ=1.45 (RC solid beams)

(27) μ=λ=0.93 (RC thin-walled hollow beams)

(28) $$\mu =\lambda =1.20\;(\mathrm{RC}\;\text{thick-walled}\;\mathrm{hollow}\;\mathrm{beams}\;\mathrm{and}\;f_{c}^{\prime }\leq 48\;\mathrm{MPa})$$

(29) $$\mu =\lambda =1.129\;(\mathrm{RC}\;\text{thick-walled}\;\mathrm{hollow}\;\mathrm{beams}\;\mathrm{and}\;f_{c}^{\prime }>48\;\mathrm{MPa})$$

The classification of the RC hollow beams into “thin wall” or “thick wall” [26] is done during the calculation procedure of the GSVATM. The RC hollow beam is firstly calculated as an equivalent RC plain beam until both the cracking torque $M_{Tcr,plain}$ and the corresponding value for the effective wall’s thickness $t_{c,cr,plain}$ are computed. Then, the following classification applies (with t being the real thickness of the wall of the RC hollow beam):

- if $t\leq 0.91t_{c,cr,plain}$ the RC hollow beam has a “thin wall”;
- if $t>0.91t_{c,cr,plain}$ the RC hollow beam has a “thick wall”.

Then, the beam is recalculated considering the real cross-section (hollow).

For the RC beams under torsion, average stresses $\sigma_{2}^{c}$ (Equation (30)) and $\sigma_{1}^{c}$ (Equation (31)) are computed for the concrete strut and tie, respectively, accounting for the section type through the correction coefficient η (Equations (32) to (35)). This simplification is assumed because the real stress diagrams along the effective wall’s thickness $t_{c}$ are not uniform due to the strain gradient (Figure 3). The coefficients $k_{2}^{c}$ and $k_{1}^{c}$ are computed from the numerical integration of the smeared σ—ε relationships.

(30) $$\sigma_{2}^{c}=\eta k_{2}^{c}\beta_{\sigma }f_{c}^{\prime }$$

(31) $$\sigma_{1}^{c}=\eta k_{1}^{c}f_{cr}$$

(32) η=1 (RC solid beams)

(33) $$\eta =0.033\sqrt{f_{c}^{\prime }\;(\mathrm{MPa})}\;+\;0.73\;(\mathrm{RC}\;\text{thin-walled}\;\mathrm{hollow}\;\mathrm{beams})$$

(34) $$\eta =0.0938\sqrt{f_{c}^{\prime }\;(\mathrm{MPa})\;}+\;0.43\;(\mathrm{RC}\;\text{thick-walled}\;\mathrm{hollow}\;\mathrm{beams}\;\mathrm{and}\;f_{c}^{\prime }\leq 48\;\mathrm{MPa})$$

(35) $$\eta =\frac{8.45}{\sqrt{f_{c}^{\prime }\;(\mathrm{MPa})}}\;+\;0.17\;(\mathrm{RC}\;\text{thick-walled}\;\mathrm{hollow}\;\mathrm{beams}\;\mathrm{and}\;f_{c}^{\prime }>48\;\mathrm{MPa})$$

**Figure 4:**
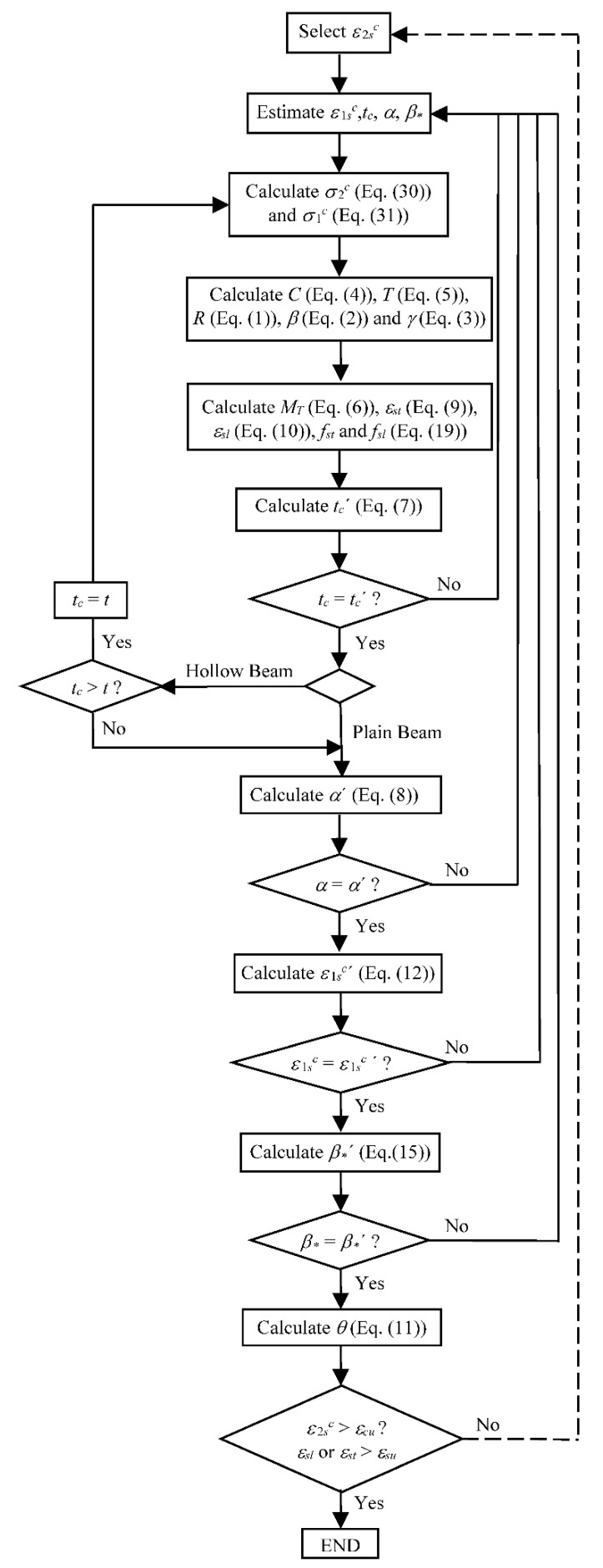
Flowchart.

To solve the nonlinear procedure of the GSVATM, an algorithm incorporating a trial-and-error technique was implemented using the programming language Delphi (see flowchart in Figure 4) [16,18]. For each iteration, the input parameter $\varepsilon_{2s}^{c}=2\varepsilon_{2}^{c}$ (strain at the outer fiber of the concrete strut) is incremented in order to compute each solution point to draw the theoretical $M_{T}$—θ curve. The calculation procedure ends when the assumed failure strains for the materials is reached, either for concrete in compression ($\varepsilon_{\mathrm{cu}}$) or for steel reinforcement in tension ($\varepsilon_{\mathrm{su}}$). In this study, European code Eurocode 2 was used to define the conventional failure strains for the materials.

## 3. Smeared Constitutive Laws for Tensile Concrete

This section presents eight smeared σ—ε relationships for tensile concrete proposed in previous studies (laws *l*1 to *l*8), so that they can be implemented in the GSVATM and checked (Section 4). In a previous study, it was showed that these relationships are suitable to be implemented in smeared truss models, such as the GSVATM, to account for the contribution of the tensile concrete [27]. 

Some of the presented smeared σ—ε relationships for tensile concrete were proposed based on the experimental results from concrete panels under shear. In such cases, the average stress $\sigma_{1}^{c}$ in the tensile concrete after cracking ($\varepsilon_{1}^{c}{\;>\;\varepsilon }_{\mathrm{cr}}$) is usually obtained from the equilibrium of the stress fields applied to the panels by separating the average stresses in both the tensile steel reinforcement and the tensile concrete. The other smeared σ—ε relationships for tensile concrete were proposed by refining the previous ones in order to improve the predictions of the used smeared models. 

For all presented smeared σ—ε relationships for tensile concrete, two equations are written. The first one aims to model the tensile behavior of the concrete before cracking and is equal for all smeared constitutive laws:

(36) $$\sigma_{1}^{c}=E_{c}\varepsilon_{1}^{c}{\;\mathrm{if}\;\varepsilon }_{1}^{c}\leq \varepsilon_{\mathrm{cr}}$$

The second equation aims to model the tensile behavior of the concrete after cracking, and accounts for the tension softening (the influence of the cracks) and the tension stiffening (the retention of concrete tensile stress due to the interaction with steel reinforcement). 

As presented in Section 2, parameters $\varepsilon_{\mathrm{cr}}$ and $E_{c}$ are computed according to Equations (24) and (25), which apply for all the presented smeared σ—ε relationships. Further, for all presented equations, the symbology was adapted to the same one used in the previous section.

### 3.1. Law l1—Cervenka in 1985 

In 1985, Cervenka proposed a smeared model for cracked RC panels. In this model, the author implemented the following equation for the descending branch of the smeared σ—ε relationships for tensile concrete [28]:

(37) $$\sigma_{1}^{c}=f_{cr}\left[1-{\left(\frac{\varepsilon_{1}^{c}}{c}\right)}^{k_{2}}\right]{\;\mathrm{if}\;\varepsilon }_{1}^{c}>\varepsilon_{\mathrm{cr}}$$

Parameter c is the average tensile strain ($\varepsilon_{1}^{c}$) for which the principal tensile stress can be considered null. The author observed that c ranges between 0.004 and 0.005. For this study, the average value (0.0045) was considered. The exponent $k_{2}$ is related with the curvature shape of the descending branch of the σ—ε curve after the peak tensile stress. Cervenka proposed to consider $k_{2}=0.5$.

### 3.2. Law l2—Vecchio and Collins in 1986

In 1986, based on several experimental results from RC panels under shear performed at the University of Toronto, Vecchio and Collins proposed the smeared model called Modified Compression Field Theory. For this model, the following postpeak smeared σ—ε relationship for tensile concrete was proposed [29]:

(38) $$\sigma_{1}^{c}=\frac{f_{cr}}{1+\sqrt{200\varepsilon_{1}^{c}}}{\;\mathrm{if}\;\varepsilon }_{1}^{c}>\varepsilon_{\mathrm{cr}}$$

### 3.3. Law l3—Hsu in 1991

In 1991, Hsu [30] proposed an efficient algorithm for his softened truss model theory to analyze the nonlinear behavior of concrete membrane elements. For this model, a refined version of the postpeak smeared σ—ε relationship for tensile concrete from Vecchio and Collins in 1986 [29] was proposed:

(39) $$\sigma_{1}^{c}=\frac{f_{cr}}{1+\sqrt{\frac{\varepsilon_{1}^{c}-\varepsilon_{\mathrm{cr}}}{0.005}}}{\;\mathrm{if}\;\varepsilon }_{1}^{c}>\varepsilon_{\mathrm{cr}}$$

### 3.4. Law l4—Belarbi and Hsu in 1994

Based on experimental studies on RC panels under shear performed at the University of Houston, Belarbi and Hsu in 1994 [24] proposed Equation (40) for the descending branch of the smeared constitutive law for tensile concrete.

(40) $$\sigma_{1}^{c}=f_{cr}{\left(\frac{\varepsilon_{\mathrm{cr}}}{\varepsilon_{1}^{c}}\right)}^{0.4}{\;\mathrm{if}\;\varepsilon }_{1}^{c}>\varepsilon_{\mathrm{cr}}$$

### 3.5. Law l5—Collins and Colaborators in 1996

In 1996, Collins et al. [31] proposed a postpeak smeared constitutive law for tensile concrete slightly different from the one proposed by Vecchio and Collins in 1986 [29]:

(41) $$\sigma_{1}^{c}=\frac{f_{cr}}{1+\sqrt{500\varepsilon_{1}^{c}}}{\;\mathrm{if}\;\varepsilon }_{1}^{c}>\varepsilon_{\mathrm{cr}}$$

### 3.6. Law l6—Vecchio in 2000

The Disturbed Stress Field Model for RC was proposed by Vecchio in 2000 [32]. For this model, the author proposed a somewhat more complicated postpeak smeared constitutive law for tensile concrete, in order to account more precisely for the tension stiffening. The author proposed two equations, with a maximum condition, to also account indirectly for the level of reinforcement ratio (Equations (42) to (45)). When a low (high) reinforcement ratio exists, tension softening (stiffening) is more relevant.

(42) $$\sigma_{1}^{c}=\max\left(f_{c1}^{a};f_{c1}^{b}\right){\;\mathrm{if}\;\varepsilon }_{1}^{c}>\varepsilon_{\mathrm{cr}}$$

(43) $$f_{c1}^{a}=f_{cr}\left(1-\frac{\varepsilon_{1}^{c}-\varepsilon_{\mathrm{cr}}}{\varepsilon_{\mathrm{ts}}-\varepsilon_{\mathrm{cr}}}\right)$$

(44) $$f_{c1}^{b}=\frac{f_{cr}}{1+\sqrt{c_{t}\varepsilon_{1}^{c}}}$$

(45) $$\varepsilon_{\mathrm{ts}}=2.0\frac{G_{f}}{f_{cr}L_{r}}$$

Parameter $\varepsilon_{\mathrm{ts}}$ represents the terminal strain, which depends on the fracture energy ($G_{f}$), assumed to be constant and equal to 75 N/m by Vecchio, and also on half of the distance between cracks ($L_{r}$). Parameter $c_{t}$ can be simply considered equal to 200 for small members or for members incorporating steel reinforcement grids with very small spacing, and 500 for large members. For this study, $L_{r}$ was infered from the experimental data of the used reference beams (Section 4).

### 3.7. Law l7—Bentz in 2005

In 2005, Bentz proposed Equations (46) and (47) for the smeared postpeak tension stiffening relationship of tensile concrete [33].

(46) $$\sigma_{1}^{c}=\frac{f_{cr}}{1+\sqrt{3.6M\varepsilon_{1}^{c}}}{\;\mathrm{if}\;\varepsilon }_{1}^{c}>\varepsilon_{\mathrm{cr}}$$

(47) $$M=\frac{A_{c}}{\sum \phi \pi }$$

Parameter M (in “mm” units) accounts for the effective tensile concrete area around the rebars ($A_{c}$) and for the rebars’ diameter (ϕ). For this study, $A_{c}$ was computed considering the effective thickness of the concrete tie ($t_{c}$), which is computed from the GSVATM.

### 3.8. Law l8—Stramandinoli and Rovere in 2008

In 2008, for the nonlinear analysis of reinforced concrete members, Stramandinoli and Rovere proposed equations for the postpeak smeared constitutive law for tensile concrete [34] (Equations (48) to (50)). The law accounts directly for the longitudinal reinforcement ratio ρ.

(48) $$\sigma_{1}^{c}=f_{cr}e^{-\alpha \left(\frac{\varepsilon_{1}^{c}}{\varepsilon_{\mathrm{cr}}}\right)}{\;\mathrm{if}\;\varepsilon }_{1}^{c}>\varepsilon_{\mathrm{cr}}$$

(49) $$\alpha =0.017+0.255\left(n\rho \right)-0.106{\left(n\rho \right)}^{2}+0.016{\left(n\rho \right)}^{3}$$

(50) $$n=\frac{E_{s}}{E_{c}}$$

### 3.9. Comparison between the Smeared Constitutive Laws

**Figure 5:**
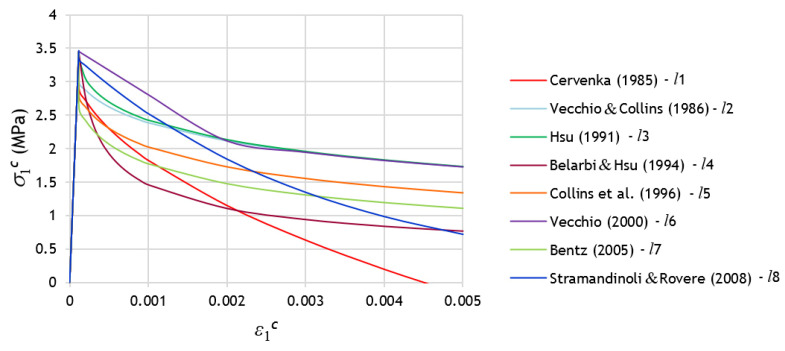
Comparison between the smeared constitutive laws.

For comparison, Figure 5 illustrates the smeared σ—ε curves for tensile concrete for each of the proposals presented in the previous subsections. The curves were computed considering the same arbitrary and typical cross-section with current materials.

**Figure 6:**
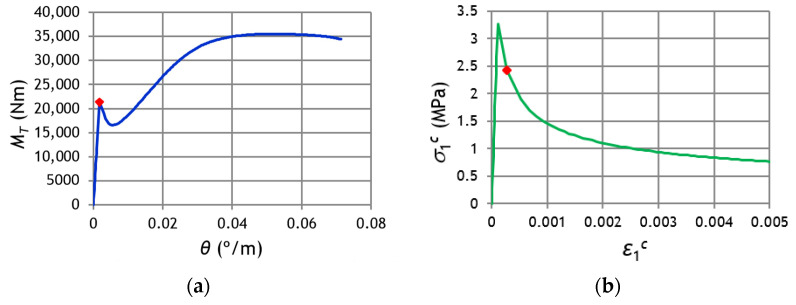
Location of the effective cracking point.

After the peak stress, namely for the descending branch, Figure 5 shows high variability between the σ—ε curves. In spite of the peak stress coincides for all the curves, it should be noted that the referred variability will influence the calculation of the cracking torque and corresponding twist with the GSVATM. This is because, as previously referred, the tensile stress $\sigma_{1}^{c}$ computed from Equation (31) represents an average stress since the real stress diagram along the effective tie’s thickness is not uniform due to the strain gradient (Figure 3). The representative concrete tensile stress in the GSVATM ($\sigma_{1}^{c}$) does not coincide with the maximum tensile stress. Hence, the strain $\varepsilon_{1}^{c}$ corresponding to the effective cracking torque in the $M_{T}$—θ curve computed with the GSVATM does not coincide with the strain $\varepsilon_{\mathrm{cr}}$ corresponding to the peak stress in the smeared σ—ε curves for tensile concrete. This is illustrated in Figure 6, where an example of $M_{T}$—θ and corresponding σ—ε curves for tensile concrete, computed with the GSVATM, are presented. The highlighted point in the curves (with marker “
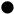
”) corresponds to the effective cracking torque, which is reached for a strain $\varepsilon_{1}^{c}$ > $\varepsilon_{\mathrm{cr}}$, i.e., in the descending branch of the smeared σ—ε curve for tensile concrete. This explains why different smeared σ—ε curves for tensile concrete incorporated in the GSVATM will lead to different coordinates for the cracking torque (cracking torque and corresponding twist). 

## 4. Comparison with Experimental Results

For this study, the experimental results of 103 RC beams tested under torsion were collected from the literature. Both RC beams with plain and hollow rectangular cross section were considered. These beams were selected based on criteria related to minimum requirements from codes of practice (for instance, the beams should incorporate a minimum torsional reinforcement, the spacing between rebars should not exceed the maximum allowed, etc.) in order to ensure a typical behavior under torsion. A detailed discussion on such applied criteria can be found in [21]. For the RC plain beams, the data were collected from the following studies: Hsu in 1968 [35], McMullen and Rangan in 1978 [36], Koutchkali and Belarbi in 2001 [37], Fang and Shiau in 2004 [38], and Peng and Wong in 2011 [39]. For RC hollow beams, the following studies were consulted: Hsu in 1968 [35], Lampert and Thürlimann in 1969 [40], Leonhardt and Schelling in 1974 [41], Bernardo and Lopes in 2009 [42], and Jeng in 2015 [26]. 

Table A1 in Appendix A summarizes the main properties for each reference beam. In Table 1, “P” and “H” stand for “plain” and “hollow” cross-section, respectively. For all the reference beams from Table A1, the experimental values of the cracking torque ($M_{Tcr}^{exp}$) and corresponding twist ($\theta_{\mathrm{cr}}^{\exp}$) were obtained from the data or graphs given by the authors [26,35,36,37,38,39,40,41,42]. Such values are presented for each reference beam in Table A2, Table A3 and Table A4 (see Appendix A). 

The torsional response of all the reference beams was computed using the GSVATM, for each of the smeared σ—ε relationships for the tensile concrete presented in Section 3 (laws *l*1 to *l*8). From the obtained theoretical $M_{T}$—θ curves, the theoretical coordinates of the cracking point, i.e., the cracking torque ($M_{Tcr}^{thli}$, with i=1 to 8) and corresponding twists, i.e., the cracking twists ($\theta_{\mathrm{cr}}^{\mathrm{thli}}$, with i=1 to 8), were obtained. Such values are also presented for each reference beam in Table A2, Table A3 and Table A4 (see Appendix A). In addition, the ratios between the experimental to the theoretical values are also presented for each reference beam ($M_{Tcr}^{exp}/M_{Tcr}^{thli}$ and $\theta_{\mathrm{cr}}^{\exp}/\theta_{\mathrm{cr}}^{\mathrm{thli}}$, with i=1 to 8).

**Figure 7:**
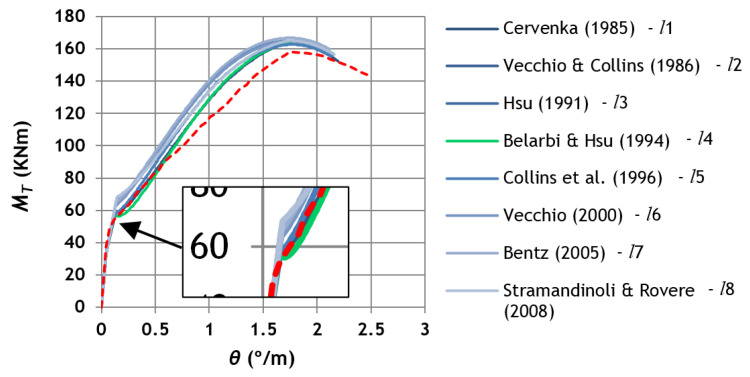
Example of $M_{T}$—θ curves for reference beam N-20-20.

Figure 7 presents, as an example, a graph with the experimental and theoretical $M_{T}$—θ curves, computed for each smeared constitutive law for tensile concrete, for reference beam N-20-20 [38]. Figure 7 confirms that the coordinates of the cracking point, namely the cracking torque, as well as the postcracking response, highly depends on the used smeared constitutive law for the tensile concrete. The influence of the used smeared constitutive law is residual at the ultimate stage, namely for the maximum torque.

Table 1 summarizes and compares the results from Table A2, Table A3 and Table A4 (Appendix A) for the cracking torque ($M_{Tcr}^{exp}$) and corresponding twist ($\theta_{\mathrm{cr}}^{\exp}$). For this, the following statistical parameters were computed for each ratio $M_{Tcr}^{exp}/M_{Tcr}^{thli}$ and $\theta_{\mathrm{cr}}^{\exp}{/\theta }_{\mathrm{cr}}^{\mathrm{thli}}$ (i=1 to 8): the average value ($\bar{x}$) and the coefficient of variation ($cv(\%)=100\times s/\bar{x}$, with s being the sample standard deviation). Table 1 also presents separately the results for plain (P) and hollow (H) beams. This is because some studies showed that noticeable differences exist between the response of plain and hollow beams under torsion for the low loading stages, namely for the transition between the uncracked and the cracked stage [26].

Table 1 shows that, for the RC plain beams, the smeared constitutive laws *l*1, *l*2, *l*4, *l*5 and *l*6 allow us to predict the cracking torque $M_{Tcr}$ (with $0.95<\bar{x}<1.05$) very well and with a very acceptable degree of dispersion (cv<13%). Among those models, the smeared constitutive law *l*4 from Belarbi and Hsu (1994) [24] is the best (with $\bar{x}=1.00$ and cv=11.35%). For the RC hollow beams, this constitutive law gives the better average value $\bar{x}=1.03$, although the degree of dispersion is high (cv=32.17%). The higher difficulty of reliably predicting the cracking torque for the RC hollow beams, when compared with the RC plain beams, was also observed and discussed in previous studies [18,26,27]. In particular, the RC hollow beams are more sensitive to the high variability of concrete tensile strength, which highly influences the cracking torque. When all beams are considered together, the smeared constitutive laws *l*2, *l*4, *l*6 and *l*7 give the best results with $\bar{x}\approx 1.00$, although the degree of dispersion is higher (cv<23%) due to the influence of the results for the RC hollow beams. In general, it can be stated that the smeared constitutive law *l*4 from Belarbi and Hsu (1994) [24] allows us to best predict the cracking torque, regardless of the cross-section type. This constitutive law has been widely used by authors in previous studies [9,16,17,18,19,23,26]. The results from Table 1 confirm the validity of such studies having chosen this smeared constitutive law for tensile concrete.

Regarding the twist corresponding to the cracking torque ($\theta_{\mathrm{cr}}$), Table 1 shows that, in general, there is a higher difficulty in obtaining a good prediction of this parameter. The constitutive laws *l*3 and *l*8 give the best average values for both the RC plain beams ($0.95<\bar{x}<1.05$) and also for all the RC beams together ($\bar{x}\leq 1.10$). However, the dispersion of these results is high (cv>25%). The results are the worst for the RC hollow beams, which was also reported in previous studies [17,18,25,27]. One possible explanation for this is that the experimental twists are very small until the end of the uncracked stage. Hence, experimental limitations related to the accurate measurement of the twists at this stage are expected. However, since the cracking twist is not very important for design, the previously reported worst results can also be considered not very important.

**Figure 8:**
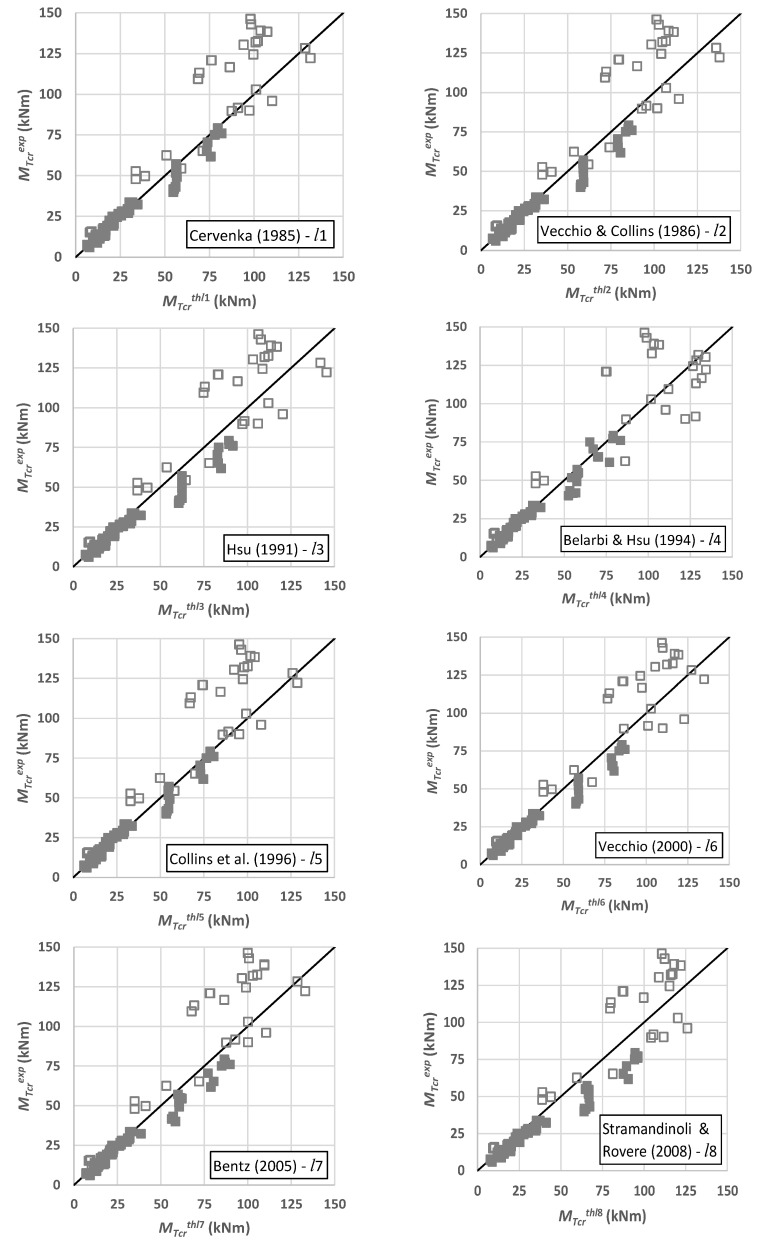
Experimental versos theoretical cracking torque.

Figure 8 presents, for each smeared constitutive law (*l*1 to *l*8), scatter graphs showing the experimental versus the theoretical values for the cracking torque. Similar graphs are not presented for the cracking twist because of the high dispersion of the results previously reported. In the graphs, different markers were used to distinguish the results regarding the cross-section type, namely “
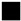
” for RC plain beams and “
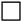
” for RC hollow beams.

Figure 8 visually confirms the observations previously stated from Table 1, namely the higher dispersion of the results for the RC hollow beams.

## 5. Conclusions

In this study, the GSVATM was used to check some proposed smeared constitutive laws for tensile concrete found in the literature in order to predict the response of the RC beams under torsion for the low loading stage; namely the transition from the uncracked stage to the cracked stage. As referred to in the introduction section, the smeared model GSVATM is simpler than the other, more complex models for the RC beams under torsion. In addition, it was also validated in several previous studies. Hence, the GSVATM was considered to be sufficiently simpler and reliable to evaluate the smeared constitutive laws for tensile concrete. From the obtained results, the following conclusions can be drawn:

(1) The different proposals for the smeared constitutive law for tensile concrete analyzed in this study lead to high differences in the shape of the postpeak descending branch of the corresponding smeared σ—ε curves;
(2) The obtained results confirm that the predicted response of the RC beams under torsion, for the transition from the uncracked stage to the cracked stage highly depends on the smeared constitutive law for tensile concrete incorporated into the model;
(3) The predictions for the cracking torque of the RC plain beams are better than the same ones for the RC hollow beams for which higher variability of the results is observed, as also reported in previous studies;
(4) Regardless of the used smeared constitutive law for tensile concrete, the cracking twist is not very well predicted. Namely, higher variability of the results is observed, as also reported in previous studies;
(5) Among the studied smeared constitutive laws for tensile concrete, the one proposed by Belarbi and Hsu in 1994 allows us to reliably predict the cracking torque of the RC beams under torsion, regardless of the cross-section type (plain or hollow). This result confirms the validity of several previous studies having incorporated this constitutive law in the used smeared truss models.

Finally, the authors consider that the results obtained in this study, using the smeared model GSVATM as reference model, can be extrapolated and could be useful to other smeared models for the RC beams under torsion. It must also be pointed out that additional solutions of experiments on the different failure mechanisms and related suitable approaches for the identification process for the parameters of relations of concrete are greatly needed and should be further studied, namely for the cracking of the RC beams under torsion.

## Figures and Tables

**Table 1:** Comparative analysis.

Cross-Section		P		H		P + H	
Constitutive law		$\frac{M_{Tcr}^{exp}}{M_{Tcr}^{thli}}$	$\frac{\theta_{\mathrm{cr}}^{\exp}}{\theta_{\mathrm{cr}}^{\mathrm{thli}}}$	$\frac{M_{Tcr}^{exp}}{M_{Tcr}^{thli}}$	$\frac{\theta_{\mathrm{cr}}^{\exp}}{\theta_{\mathrm{cr}}^{\mathrm{thli}}}$	$\frac{M_{Tcr}^{exp}}{M_{Tcr}^{thli}}$	$\frac{\theta_{\mathrm{cr}}^{\exp}}{\theta_{\mathrm{cr}}^{\mathrm{thli}}}$
*l*1—Cervenka (1985) [28]	$\bar{x}=$	1.02	1.16	1.29	1.71	1.05	1.23
	cv(%)=	12.13	25.19	21.47	35.22	21.29	38.54
*l*2—Vecchio and Collins (1986) [29]	$\bar{x}=$	0.96	1.11	1.24	1.62	0.99	1.16
	cv(%)=	12.41	25.68	21.47	35.61	21.73	39.13
*l*3—Hsu (1991) [30]	$\bar{x}=$	0.91	1.04	1.18	1.55	0.94	1.10
	cv(%)=	12.38	25.83	21.66	36.11	22.13	40.05
*l*4—Belarbi and Hsu (1994) [24]	$\bar{x}=$	1.00	1.16	1.03	1.42	1.01	1.19
	cv(%)=	11.35	24.10	32.17	46.05	21.45	39.02
*l*5—Collins et al. (1996) [31]	$\bar{x}=$	1.04	1.20	1.33	1.74	1.07	1.24
	cv(%)=	12.31	25.29	21.49	35.97	21.36	39.23
*l*6—Vecchio (2000) [32]	$\bar{x}=$	0.96	1.08	1.18	1.50	1.00	1.15
	cv(%)=	11.82	26.20	19.22	37.88	18.56	37.43
*l*7—Bentz (2005) [33]	$\bar{x}=$	0.94	1.08	1.26	1.68	0.99	1.16
	cv(%)=	12.24	25.91	21.23	34.90	22.79	40.35
*l*8—Stramandinoli and Rovere (2008) [34]	$\bar{x}=$	0.86	0.98	1.13	1.46	0.89	1.06
	cv(%)=	12.84	26.75	22.11	36.09	22.72	39.36

## Data Availability

Data is contained within the article.

## Appendix A

**Table A1:** Properties of the reference beams.

Beam		x cm	y cm	t cm	$x_{1}$ cm	$y_{1}$ cm	$A_{st}/s$ cm^2^/m	$A_{sl}$ cm^2^	$\rho_{t}$ %	$\rho_{l}$ %	$f_{ty}$ MPa	$f_{ly}$ MPa	${f^{\prime }}_{c}$ MPa	$\varepsilon_{ο}$ %
B3 [35]	P	25.4	38.1	-	21.6	34.3	10.16	11.36	1.17	1.17	320	328	28.1	0.20
B4 [35]	P	25.4	38.1	-	21.6	34.3	14.01	15.48	1.62	1.60	323	320	29.2	0.20
B5 [35]	P	25.4	38.1	-	21.6	34.3	18.47	20.39	2.13	2.11	321	332	30.6	0.20
B6 [35]	P	25.4	38.1	-	21.6	34.3	22.58	25.81	2.61	2.67	323	332	28.8	0.20
B7 [35]	P	25.4	38.1	-	21.6	34.3	10.16	5.16	1.17	0.53	319	320	26.0	0.19
B8 [35]	P	25.4	38.1	-	21.6	34.3	22.58	5.16	2.61	0.53	320	322	26.8	0.19
B9 [35]	P	25.4	38.1	-	21.6	34.3	4.66	11.36	0.54	1.17	343	319	28.8	0.20
C4 [35]	P	25.4	25.4	-	21.6	21.6	13.11	11.36	1.76	1.76	328	337	27.2	0.20
C5 [35]	P	25.4	25.4	-	21.6	21.6	17.67	15.48	2.37	2.40	329	328	27.2	0.20
C6 [35]	P	25.4	25.4	-	21.6	21.6	23.91	20.39	3.20	3.16	328	316	27.6	0.20
G3 [35]	P	25.4	50.8	-	21.6	47.0	8.29	11.36	0.88	0.88	328	339	26.8	0.19
G4 [35]	P	25.4	50.8	-	21.6	47.0	11.29	15.48	1.20	1.20	321	326	28.3	0.20
G5 [35]	P	25.4	50.8	-	21.6	47.0	15.05	20.39	1.60	1.58	328	331	26.9	0.19
G7 [35]	P	25.4	50.8	-	21.6	47.0	8.84	12.00	0.94	0.93	323	319	31.0	0.20
G8 [35]	P	25.4	50.8	-	21.6	47.0	12.32	17.03	1.31	1.32	329	322	28.3	0.20
I3 [35]	P	25.4	38.1	-	21.6	34.3	10.16	11.36	1.17	1.17	334	343	44.8	0.23
I4 [35]	P	25.4	38.1	-	21.6	34.3	14.01	15.48	1.62	1.60	326	315	45.0	0.23
I5 [35]	P	25.4	38.1	-	21.6	34.3	18.47	20.39	2.13	2.11	326	310	45.0	0.23
I6 [35]	P	25.4	38.1	-	21.6	34.3	22.58	25.81	2.61	2.67	329	326	45.8	0.23
J1 [35]	P	25.4	38.1	-	21.6	34.3	4.66	5.16	0.54	0.53	346	328	14.3	0.16
J2 [35]	P	25.4	38.1	-	21.6	34.3	7.21	8.00	0.83	0.83	341	320	14.6	0.16
J3 [35]	P	25.4	38.1	-	21.6	34.3	10.16	11.36	1.17	1.17	337	389	16.9	0.17
J4 [35]	P	25.4	38.1	-	21.6	34.3	14.01	15.48	1.62	1.60	332	324	16.8	0.17
K2 [35]	P	15.2	49.5	-	11.4	45.7	6.77	7.74	1.03	1.03	338	336	30.6	0.20
K3 [35]	P	15.2	49.5	-	11.4	45.7	10.42	12.00	1.58	1.59	321	316	29.0	0.20
K4 [35]	P	15.2	49.5	-	11.4	45.7	15.05	17.03	2.28	2.26	340	344	28.6	0.20
M1 [35]	P	25.4	38.1	-	21.6	34.3	4.76	8.00	0.55	0.83	353	326	29.9	0.20
M2 [35]	P	25.4	38.1	-	21.6	34.3	6.77	11.36	0.78	1.17	357	329	30.6	0.20
M3 [35]	P	25.4	38.1	-	21.6	34.3	9.24	15.48	1.07	1.60	326	322	26.8	0.29
M4 [35]	P	25.4	38.1	-	21.6	34.3	12.33	20.39	1.42	2.11	327	319	26.6	0.19
M5 [35]	P	25.4	38.1	-	21.6	34.3	15.63	25.81	1.81	2.67	331	335	28.0	0.20
M6 [35]	P	25.4	38.1	-	21.6	34.3	15.63	30.58	1.81	3.16	341	318	29.4	0.20
N1 [35]	P	15.2	30.5	-	13.0	28.3	3.50	2.84	0.62	0.61	341	352	29.5	0.20
N1a [35]	P	15.2	30.5	-	13.0	28.3	3.50	2.84	0.62	0.61	345	346	28.7	0.20
N2 [35]	P	15.2	30.5	-	13.0	28.3	6.35	5.16	1.13	1.11	338	331	30.4	0.20
N2a [35]	P	15.2	30.5	-	13.0	28.3	6.21	1.61	1.10	1.11	361	333	28.4	0.20
N3 [35]	P	15.2	30.5	-	13.0	28.3	5.08	4.26	0.90	0.92	352	352	27.3	0.20
N4 [35]	P	15.2	30.5	-	13.0	28.3	7.98	6.58	1.42	1.42	356	341	27.3	0.20
A2 [36]	P	25.4	25.4	-	22.2	22.2	7.82	5.16	1.08	0.80	285	380	38.2	0.22
A3 [36]	P	25.4	25.4	-	21.9	21.9	8.94	8.00	1.22	1.24	360	352	39.4	0.22
A4 [36]	P	25.4	25.4	-	21.9	21.9	12.42	11.36	1.69	1.76	360	351	39.2	0.22
B3 [36]	P	17.8	35.6	-	14.3	32.1	8.60	8.00	1.26	1.27	360	352	38.6	0.22
B4 [36]	P	17.8	35.6	-	14.3	32.1	11.76	11.36	1.73	1.80	360	351	38.5	0.22
B5UR1 [37]	P	20.3	30.5	-	16.5	26.7	6.56	5.16	0.92	0.83	373	386	39.6	0.20
B9UR1 [37]	P	20.3	30.5	-	16.5	26.7	6.56	5.16	0.92	0.83	373	386	75.0	0.27
B12UR1 [37]	P	20.3	30.5	-	16.5	26.7	6.56	5.16	0.92	0.83	399	386	80.6	0.27
B14UR1 [37]	P	20.3	30.5	-	16.5	26.7	6.56	5.16	0.92	0.83	386	386	93.9	0.29
B12UR2 [37]	P	20.3	30.5	-	16.5	26.7	6.95	5.16	0.97	0.83	386	386	76.2	0.27
B12UR3 [37]	P	20.3	30.5	-	16.5	26.7	7.46	6.58	1.04	1.06	386	380	72.9	0.26
B12UR4 [37]	P	20.3	30.5	-	16.5	26.7	7.88	7.74	1.10	1.25	386	373	75.9	0.27
B12UR5 [37]	P	20.3	30.5	-	16.5	26.7	10.13	8.00	1.41	1.29	386	380	76.7	0.27
H-06-12 [38]	P	35.0	50.0	-	30.0	45.0	7.10	20.65	0.61	1.18	440	410	78.5	0.27
H-07-10 [38]	P	35.0	50.0	-	30.0	45.0	7.89	17.03	0.68	0.97	420	500	68.4	0.26
H-07-16 [38]	P	35.0	50.0	-	30.0	45.0	7.89	28.39	0.68	1.62	420	500	68.4	0.26
H-12-12 [38]	P	35.0	50.0	-	30.0	45.0	14.19	20.65	1.22	1.18	440	410	78.5	0.27
H-12-16 [38]	P	35.0	50.0	-	30.0	45.0	14.19	28.39	1.22	1.62	440	520	78.5	0.27
H-14-10 [38]	P	35.0	50.0	-	30.0	45.0	16.13	17.03	1.38	0.97	360	500	68.4	0.26
H-20-20 [38]	P	35.0	50.0	-	30.0	45.0	23.46	34.06	2.01	1.95	440	560	78.5	0.27
N-06-06 [38]	P	35.0	50.0	-	30.0	45.0	7.10	12.00	0.61	0.69	440	440	35.5	0.21
N-06-12 [38]	P	35.0	50.0	-	30.0	45.0	7.10	20.65	0.61	1.18	440	410	35.5	0.21
N-07-10 [38]	P	35.0	50.0	-	30.0	45.0	7.89	17.03	0.68	0.97	420	500	33.5	0.21
N-07-16 [38]	P	35.0	50.0	-	30.0	45.0	7.89	28.39	0.68	1.62	420	500	33.5	0.21
N-12-12 [38]	P	35.0	50.0	-	30.0	45.0	14.19	20.65	1.22	1.18	440	410	35.5	0.21
N-12-16 [38]	P	35.0	50.0	-	30.0	45.0	14.19	28.39	1.22	1.62	440	520	35.5	0.21
N-14-10 [38]	P	35.0	50.0	-	30.0	45.0	16.13	17.03	1.38	0.97	360	500	33.5	0.21
N-20-20 [38]	P	35.0	50.0	-	30.0	45.0	23.46	34.06	2.01	1.95	440	560	35.5	0.21
SW12-1 [39]	P	15.0	120.0	-	10.0	115.0	3.93	11.31	0.55	1.26	459	480	44.2	0.23
SW10-1 [39]	P	15.0	100.0	-	10.0	95.0	3.93	9.05	0.55	1.21	459	499	29.5	0.20
SW10-2 [39]	P	15.0	100.0	-	9.8	94.8	7.54	9.05	1.05	1.21	480	480	44.2	0.23
SW10-3 [39]	P	15.0	100.0	-	9.8	94.8	11.31	9.05	1.58	1.21	499	499	29.5	0.20
SW10-4 [39]	P	15.0	100.0	-	9.4	94.4	16.08	16.08	2.23	2.14	497	497	33.8	0.21
SW8-1 [39]	P	15.0	80.0	-	10.2	75.2	4.02	7.07	0.57	1.18	433	459	29.5	0.20
SW8-2 [39]	P	15.0	80.0	-	9.8	74.8	11.31	7.07	1.59	1.18	499	459	29.5	0.20
D3 [35]	H	25.4	38.1	6.4	21.6	34.3	10.16	11.36	1.17	1.17	333	341	28.4	0.20
D4 [35]	H	25.4	38.1	6.4	21.6	34.3	14.01	15.48	1.62	1.60	333	330	30.6	0.20
T0 [40]	H	50.0	50.0	8.0	43.0	43.0	10.28	32.16	0.71	1.29	357	345	45.1	0.23
T1 [40]	H	50.0	50.0	8.0	45.4	45.4	10.28	18.10	0.75	0.72	357	357	35.3	0.21
T2 [40]	H	50.0	50.0	8.0	43.0	43.0	10.28	18.10	0.71	0.72	357	357	35.3	0.21
T5 [40]	H	80.0	40.0	8.0	73.0	33.0	10.28	10.00	0.68	0.31	513	529	47.1	0.23
VH1 [41]	H	32.4	32.4	8.0	30.4	30.4	2.88	3.46	0.33	0.33	447	447	17.2	0.17
VH2 [41]	H	32.4	32.4	8.0	30.4	30.4	5.76	6.91	0.67	0.66	447	447	17.2	0.17
A2 [42]	H	60.0	60.0	10.7	53.8	53.1	6.28	13.95	0.37	0.39	696	672	47.3	0.23
A3 [42]	H	60.0	60.0	10.9	53.5	53.5	8.27	18.10	0.49	0.50	715	672	46.2	0.23
A4 [42]	H	60.0	60.0	10.4	52.0	52.5	11.22	23.75	0.65	0.66	715	724	54.8	0.24
A5 [42]	H	60.0	60.0	10.4	52.8	52.8	14.14	30.66	0.83	0.85	672	724	53.1	0.24
B2 [42]	H	60.0	60.0	10.8	53.3	53.4	6.70	14.58	0.40	0.41	696	672	69.8	0.26
B3 [42]	H	60.0	60.0	10.9	53.5	53.7	11.22	23.75	0.67	0.66	715	724	77.8	0.27
B4 [42]	H	60.0	60.0	11.2	52.3	53.6	15.08	32.17	0.89	0.89	672	724	79.8	0.27
B5 [42]	H	60.0	60.0	11.7	51.8	51.8	18.85	40.21	1.09	1.12	672	724	76.4	0.27
C2 [42]	H	60.0	60.0	10.0	53.2	53.3	6.28	13.95	0.37	0.39	696	672	94.8	0.28
C3 [42]	H	60.0	60.0	10.3	54.5	54.0	10.47	23.75	0.63	0.66	715	724	91.6	0.28
C4 [42]	H	60.0	60.0	10.3	54.6	54.5	14.14	30.66	0.86	0.85	672	724	91.4	0.28
C5 [42]	H	60.0	60.0	10.4	54.0	54.3	17.40	36.69	1.05	1.02	672	724	96.7	0.28
C6 [42]	H	60.0	60.0	10.4	53.3	52.9	22.62	48.25	1.34	1.34	672	724	87.5	0.28
A095c [26]	H	49.7	71.1	14.5	43.7	65.1	9.93	13.16	0.61	0.37	381	371	35.1	0.21
A120a [26]	H	50.2	71.9	18.4	44.2	65.9	7.59	20.00	0.46	0.55	380	464	27.6	0.20
B065b [26]	H	50.3	71.0	9.2	44.3	65.0	9.93	50.97	0.61	1.43	380	452	39.2	0.22
B080a [26]	H	50.0	72.1	11.2	44.0	66.1	12.90	28.39	0.79	0.79	392	454	46.5	0.23
B110a [26]	H	49.8	71.0	15.5	43.8	65.0	8.60	20.00	0.53	0.57	369	453	48.1	0.23
C065a [26]	H	49.5	78.1	8.5	43.5	72.1	9.93	20.00	0.59	0.52	376	338	78.8	0.27
C100a [26]	H	49.9	72.3	12.7	43.9	66.3	12.90	28.39	0.79	0.79	447	466	90.6	0.28
D075a [26]	H	49.8	73.4	8.7	43.8	67.4	12.90	28.39	0.79	0.78	381	469	94.9	0.29
D090a [26]	H	50.1	72.2	10.5	44.1	66.2	12.90	28.39	0.79	0.79	447	466	105.7	0.30

**Table A2:** Cracking torques and corresponding twists (smeared constitutive laws *l*1 to *l*3).

Beam	$M_{Tcr}^{exp}$ kNm	$\theta_{\mathrm{cr}}^{\exp}$ °/m	$M_{Tcr}^{thl1}$ kNm	$\frac{M_{Tcr}^{exp}}{M_{Tcr}^{thl1}}$	$\theta_{\mathrm{cr}}^{\mathrm{thl}1}$ °/m	$\frac{\theta_{\mathrm{cr}}^{\exp}}{\theta_{\mathrm{cr}}^{\mathrm{thl}1}}$	$M_{Tcr}^{thl2}$ kNm	$\frac{M_{Tcr}^{exp}}{M_{Tcr}^{thl2}}$	$\theta_{\mathrm{cr}}^{\mathrm{thl}2}$ °/m	$\frac{\theta_{\mathrm{cr}}^{\exp}}{\theta_{\mathrm{cr}}^{\mathrm{thl}2}}$	$M_{Tcr}^{thl3}$ kNm	$\frac{M_{Tcr}^{exp}}{M_{Tcr}^{thl3}}$	$\theta_{\mathrm{cr}}^{\mathrm{thl}3}$ °/m	$\frac{\theta_{\mathrm{cr}}^{\exp}}{\theta_{\mathrm{cr}}^{\mathrm{thl}3}}$
B3 [35]	20.1	0.12	20.9	0.96	0.10	1.21	22.0	0.91	0.11	1.15	23.2	0.87	0.11	1.09
B4 [35]	21.9	0.12	21.0	1.05	0.10	1.20	22.0	0.99	0.10	1.15	23.1	0.95	0.11	1.09
B5 [35]	22.6	0.14	21.4	1.05	0.10	1.42	22.2	1.02	0.10	1.36	23.4	0.97	0.11	1.30
B6 [35]	25.0	0.16	20.6	1.21	0.09	1.75	21.7	1.15	0.10	1.67	22.8	1.09	0.10	1.58
B7 [35]	20.2	0.11	20.0	1.01	0.10	1.07	21.0	0.96	0.11	1.02	22.1	0.91	0.11	0.97
B8 [35]	21.8	0.13	20.3	1.07	0.10	1.28	21.3	1.02	0.10	1.22	22.3	0.98	0.11	1.17
B9 [35]	19.6	0.11	20.8	0.94	0.10	1.04	22.0	0.89	0.11	0.99	23.2	0.85	0.11	0.94
C4 [35]	11.9	0.13	11.3	1.05	0.11	1.18	11.8	1.01	0.12	1.12	12.4	0.96	0.13	1.07
C5 [35]	14.0	0.17	11.2	1.25	0.11	1.51	11.9	1.17	0.12	1.41	12.5	1.12	0.12	1.35
C6 [35]	13.9	0.17	11.3	1.23	0.11	1.61	11.5	1.20	0.11	1.57	12.0	1.15	0.11	1.51
G3 [35]	27.1	0.10	29.5	0.92	0.09	1.05	31.0	0.87	0.10	1.00	32.7	0.83	0.10	0.95
G4 [35]	28.7	0.12	30.1	0.95	0.09	1.29	31.6	0.91	0.10	1.23	33.4	0.86	0.10	1.16
G5 [35]	29.5	0.11	29.2	1.01	0.09	1.30	30.7	0.96	0.09	1.24	32.3	0.91	0.10	1.17
G7 [35]	33.6	0.13	31.7	1.06	0.09	1.45	33.3	1.01	0.10	1.38	35.1	0.96	0.10	1.31
G8 [35]	33.6	0.12	30.1	1.12	0.09	1.37	31.6	1.06	0.09	1.30	33.4	1.01	0.10	1.23
I3 [35]	25.5	0.11	25.5	1.00	0.11	0.97	27.2	0.94	0.12	0.91	28.7	0.89	0.12	0.86
I4 [35]	28.0	0.12	25.7	1.09	0.11	1.15	27.3	1.03	0.11	1.08	28.8	0.97	0.12	1.02
I5 [35]	28.1	0.15	26.0	1.08	0.11	1.43	27.4	1.02	0.11	1.36	28.9	0.97	0.12	1.29
I6 [35]	27.5	0.13	26.2	1.05	0.10	1.22	27.7	0.99	0.11	1.15	29.2	0.94	0.12	1.09
J1 [35]	14.0	0.09	15.3	0.92	0.09	0.92	15.9	0.88	0.10	0.88	16.5	0.85	0.10	0.85
J2 [35]	17.1	0.12	15.1	1.13	0.09	1.38	15.7	1.09	0.09	1.32	16.5	1.03	0.10	1.26
J3 [35]	16.9	0.10	15.9	1.06	0.09	1.08	16.6	1.02	0.09	1.03	17.5	0.97	0.10	0.98
J4 [35]	18.0	0.11	15.6	1.15	0.09	1.28	16.3	1.10	0.09	1.23	17.1	1.05	0.09	1.17
K2 [35]	12.2	0.18	12.0	1.02	0.14	1.31	12.3	0.99	0.14	1.27	13.1	0.93	0.15	1.20
K3 [35]	12.4	0.19	11.5	1.08	0.13	1.49	11.9	1.05	0.13	1.45	12.6	0.98	0.14	1.37
K4 [35]	13.1	0.21	11.1	1.19	0.12	1.71	11.6	1.13	0.13	1.63	12.4	1.06	0.13	1.53
M1 [35]	19.2	0.11	21.2	0.90	0.10	1.03	22.5	0.85	0.11	0.97	23.6	0.81	0.12	0.92
M2 [35]	20.6	0.11	21.5	0.96	0.10	1.08	22.5	0.92	0.11	1.03	23.8	0.86	0.11	0.97
M3 [35]	20.7	0.12	20.0	1.03	0.10	1.24	21.0	0.98	0.10	1.18	22.2	0.93	0.11	1.12
M4 [35]	20.7	0.13	19.9	1.04	0.10	1.41	20.8	0.99	0.10	1.34	22.0	0.94	0.11	1.27
M5 [35]	21.7	0.12	20.2	1.07	0.09	1.30	21.3	1.02	0.10	1.23	22.4	0.97	0.10	1.17
M6 [35]	22.7	0.15	20.7	1.10	0.09	1.57	21.7	1.05	0.10	1.49	22.8	0.99	0.10	1.42
N1 [35]	7.6	0.13	6.7	1.14	0.16	0.81	7.0	1.08	0.17	0.77	7.4	1.02	0.18	0.73
N1a [35]	7.0	0.11	6.6	1.06	0.16	0.69	6.9	1.01	0.17	0.66	7.3	0.96	0.17	0.62
N2 [35]	7.4	0.22	6.8	1.10	0.15	1.40	7.1	1.05	0.16	1.34	7.5	0.99	0.17	1.27
N2a [35]	7.5	0.21	6.6	1.14	0.15	1.37	6.9	1.09	0.16	1.31	7.3	1.03	0.17	1.24
N3 [35]	7.4	0.21	6.4	1.15	0.15	1.39	6.7	1.10	0.16	1.33	7.1	1.04	0.17	1.25
N4 [35]	7.6	0.21	6.4	1.19	0.15	1.43	6.7	1.13	0.16	1.36	7.1	1.07	0.16	1.29
A2 [36]	11.3	0.12	13.4	0.84	0.13	0.94	14.0	0.81	0.13	0.89	14.8	0.76	0.14	0.84
A3 [36]	12.2	0.12	13.5	0.90	0.13	0.98	14.3	0.85	0.13	0.92	15.1	0.81	0.14	0.88
A4 [36]	12.5	0.15	13.5	0.93	0.12	1.20	14.3	0.88	0.13	1.14	15.1	0.83	0.14	1.08
B3 [36]	8.8	0.15	12.0	0.73	0.14	1.06	12.6	0.70	0.14	1.01	13.3	0.66	0.15	0.96
B4 [36]	10.2	0.15	12.0	0.85	0.13	1.09	12.6	0.81	0.14	1.04	13.3	0.77	0.15	0.98
B5UR1 [37]	11.6	0.09	12.4	0.94	0.14	0.63	13.1	0.89	0.14	0.60	13.8	0.84	0.15	0.57
B9UR1 [37]	13.0	0.13	16.2	0.80	0.15	0.91	17.3	0.75	0.16	0.86	18.2	0.71	0.17	0.81
B12UR1 [37]	16.2	0.09	16.7	0.97	0.15	0.61	17.8	0.91	0.16	0.57	18.8	0.86	0.17	0.55
B14UR1 [37]	19.3	0.12	17.7	1.09	0.15	0.78	18.9	1.02	0.16	0.73	19.9	0.97	0.17	0.69
B12UR2 [37]	17.8	0.11	16.4	1.09	0.15	0.75	17.4	1.02	0.16	0.71	18.4	0.97	0.17	0.67
B12UR3 [37]	16.0	0.10	16.1	1.00	0.15	0.70	17.3	0.93	0.16	0.65	18.0	0.89	0.16	0.62
B12UR4 [37]	16.9	0.14	16.4	1.03	0.15	0.96	17.5	0.96	0.16	0.89	18.4	0.92	0.16	0.85
B12UR5 [37]	13.6	0.04	16.7	0.81	0.15	0.24	17.8	0.76	0.16	0.23	18.6	0.73	0.16	0.22
H-06-12 [38]	75.0	0.09	78.0	0.96	0.09	1.00	83.5	0.90	0.09	0.93	83.5	0.90	0.09	0.93
H-07-10 [38]	70.5	0.09	73.9	0.95	0.09	1.08	79.0	0.89	0.09	1.01	82.9	0.85	0.10	0.96
H-07-16 [38]	65.3	0.09	73.5	0.89	0.08	1.03	79.2	0.82	0.09	0.95	83.1	0.79	0.09	0.91
H-12-12 [38]	77.1	0.07	79.7	0.97	0.09	0.85	85.0	0.91	0.09	0.80	89.5	0.86	0.10	0.76
H-12-16 [38]	79.3	0.09	79.7	1.00	0.09	1.06	85.3	0.93	0.09	0.99	89.4	0.89	0.10	0.95
H-14-10 [38]	61.8	0.09	75.7	0.82	0.09	1.00	80.6	0.77	0.09	0.94	84.9	0.73	0.10	0.89
H-20-20 [38]	76.0	0.09	81.8	0.93	0.09	1.05	87.1	0.87	0.09	0.99	91.6	0.83	0.10	0.94
N-06-06 [38]	43.2	0.08	56.1	0.77	0.08	1.02	59.3	0.73	0.08	0.96	62.5	0.69	0.09	0.91
N-06-12 [38]	51.8	0.11	56.1	0.92	0.08	1.47	59.1	0.88	0.08	1.39	62.4	0.83	0.09	1.32
N-07-10 [38]	41.6	0.11	54.7	0.76	0.08	1.40	57.6	0.72	0.08	1.33	60.7	0.68	0.09	1.26
N-07-16 [38]	40.0	0.11	54.7	0.73	0.08	1.43	57.5	0.70	0.08	1.36	60.6	0.66	0.08	1.29
N-12-12 [38]	49.3	0.09	56.7	0.87	0.08	1.18	59.2	0.83	0.08	1.13	62.5	0.79	0.09	1.07
N-12-16 [38]	57.1	0.12	56.3	1.02	0.08	1.58	59.2	0.96	0.08	1.50	62.5	0.91	0.08	1.42
N-14-10 [38]	41.8	0.12	55.2	0.76	0.08	1.56	57.9	0.72	0.08	1.49	61.1	0.68	0.09	1.41
N-20-20 [38]	55.0	0.13	56.6	0.97	0.08	1.68	58.9	0.93	0.08	1.61	62.4	0.88	0.08	1.52
SW12-1 [39]	32.3	0.15	34.6	0.93	0.13	1.16	36.4	0.89	0.14	1.11	38.8	0.83	0.15	1.03
SW10-1 [39]	24.6	0.13	23.2	1.06	0.13	1.00	24.7	1.00	0.14	0.93	25.8	0.95	0.14	0.90
SW10-2 [39]	29.6	0.20	29.1	1.02	0.14	1.47	31.1	0.95	0.15	1.37	32.0	0.92	0.15	1.36
SW10-3 [39]	26.6	0.15	23.9	1.11	0.13	1.16	25.3	1.05	0.14	1.10	26.7	1.00	0.15	1.04
SW10-4 [39]	27.7	0.16	25.7	1.08	0.13	1.24	27.1	1.02	0.14	1.17	28.8	0.96	0.15	1.10
SW8-1 [39]	19.7	0.16	18.6	1.06	0.14	1.13	20.0	0.98	0.15	1.03	21.0	0.94	0.16	0.99
SW8-2 [39]	22.5	0.14	18.9	1.19	0.14	1.06	20.1	1.12	0.15	0.98	21.2	1.06	0.15	0.94
D3 [35]	15.2	0.08	8.0	1.89	0.05	1.49	8.5	1.79	0.06	1.40	8.8	1.73	0.06	1.36
D4 [35]	15.8	0.12	8.9	1.78	0.06	2.05	9.3	1.70	0.06	1.96	9.7	1.63	0.06	1.88
T0 [40]	49.8	0.06	38.8	1.28	0.03	1.92	40.8	1.22	0.03	1.81	42.5	1.17	0.04	1.75
T1 [40]	48.0	0.04	33.7	1.43	0.03	1.35	35.3	1.36	0.03	1.27	36.8	1.31	0.03	1.23
T2 [40]	52.8	0.10	33.7	1.57	0.03	3.07	35.3	1.49	0.03	2.90	36.8	1.44	0.03	2.81
T5 [40]	62.5	0.06	50.8	1.23	0.03	2.07	53.7	1.16	0.03	1.89	53.6	1.17	0.03	2.10
VH1 [41]	12.0	0.12	9.8	1.22	0.07	1.65	10.4	1.15	0.08	1.55	10.9	1.10	0.08	1.48
VH2 [41]	11.5	0.07	10.4	1.10	0.08	0.90	11.1	1.03	0.08	0.85	11.7	0.99	0.09	0.81
A2 [42]	109.5	0.06	68.6	1.60	0.03	2.44	71.6	1.53	0.03	2.34	74.8	1.46	0.03	2.25
A3 [42]	113.3	0.06	69.2	1.64	0.03	2.18	72.3	1.57	0.03	2.09	75.5	1.50	0.03	2.02
A4 [42]	120.9	0.06	75.9	1.59	0.03	2.42	79.8	1.51	0.03	2.25	83.4	1.45	0.03	2.17
A5 [42]	120.9	0.04	76.1	1.59	0.03	1.66	79.4	1.52	0.03	1.59	82.9	1.46	0.03	1.53
B2 [42]	116.7	0.04	86.3	1.35	0.03	1.69	90.2	1.29	0.03	1.60	94.4	1.24	0.03	1.53
B3 [42]	130.5	0.05	94.2	1.39	0.03	1.74	98.4	1.33	0.03	1.65	103.1	1.26	0.03	1.57
B4 [42]	142.9	0.07	98.3	1.45	0.03	2.73	102.7	1.39	0.03	2.58	107.7	1.33	0.03	2.47
B5 [42]	146.3	0.06	98.0	1.49	0.03	2.44	101.4	1.44	0.03	2.38	106.3	1.38	0.03	2.28
C2 [42]	124.5	0.05	99.6	1.25	0.03	1.81	104.1	1.20	0.03	1.70	108.6	1.15	0.03	1.67
C3 [42]	131.9	0.06	100.8	1.31	0.03	2.35	104.7	1.26	0.03	2.29	109.9	1.20	0.03	2.18
C4 [42]	132.6	0.05	102.1	1.30	0.03	1.92	106.8	1.24	0.03	1.82	112.0	1.18	0.03	1.73
C5 [42]	138.3	0.05	107.4	1.29	0.03	1.91	111.3	1.24	0.03	1.85	116.9	1.18	0.03	1.76
C6 [42]	139.1	0.05	103.7	1.34	0.03	2.02	108.3	1.28	0.03	1.91	113.5	1.23	0.03	1.83
A095c [26]	102.9	0.03	101.0	1.02	0.04	0.82	106.9	0.96	0.04	0.77	112.0	0.92	0.05	0.74
A120a [26]	89.8	0.05	87.4	1.03	0.04	1.14	92.8	0.97	0.04	1.07	97.1	0.92	0.05	1.03
B065b [26]	54.4	0.03	59.1	0.92	0.03	1.23	62.1	0.88	0.03	1.16	64.7	0.84	0.03	1.12
B080a [26]	65.2	0.03	71.2	0.92	0.03	1.24	74.2	0.88	0.03	1.19	78.0	0.84	0.03	1.12
B110a [26]	128.3	0.04	128.6	1.00	0.04	0.99	135.8	0.94	0.04	0.93	141.8	0.90	0.05	0.90
C065a [26]	91.7	0.03	90.9	1.01	0.03	1.06	95.5	0.96	0.03	0.98	98.4	0.93	0.03	1.03
C100a [26]	122.2	0.03	131.6	0.93	0.03	0.85	137.8	0.89	0.04	0.81	145.3	0.84	0.04	0.76
D075a [26]	90.1	0.03	97.3	0.93	0.03	0.99	101.8	0.88	0.03	0.94	106.0	0.85	0.03	0.92
D090a [26]	96.1	0.03	110.0	0.87	0.03	1.08	114.3	0.84	0.03	1.05	120.2	0.80	0.03	0.99

**Table A3:** Cracking torques and corresponding twists (smeared constitutive laws *l*4 to *l*6).

Beam	$M_{Tcr}^{exp}$ kNm	$\theta_{\mathrm{cr}}^{\exp}$ °/m	$M_{Tcr}^{thl4}$ kNm	$\frac{M_{Tcr}^{exp}}{M_{Tcr}^{thl4}}$	$\theta_{\mathrm{cr}}^{\mathrm{thl}4}$ °/m	$\frac{\theta_{\mathrm{cr}}^{\exp}}{\theta_{\mathrm{cr}}^{\mathrm{thl}4}}$	$M_{Tcr}^{thl5}$ kNm	$\frac{M_{Tcr}^{exp}}{M_{Tcr}^{thl5}}$	$\theta_{\mathrm{cr}}^{\mathrm{thl}5}$ °/m	$\frac{\theta_{\mathrm{cr}}^{\exp}}{\theta_{\mathrm{cr}}^{\mathrm{thl}5}}$	$M_{Tcr}^{thl6}$ kNm	$\frac{M_{Tcr}^{exp}}{M_{Tcr}^{thl6}}$	$\theta_{\mathrm{cr}}^{\mathrm{thl}6}$ °/m	$\frac{\theta_{\mathrm{cr}}^{\exp}}{\theta_{\mathrm{cr}}^{\mathrm{thl}6}}$
B3 [35]	20.1	0.12	21.3	0.94	0.10	1.19	20.4	0.99	0.10	1.24	22.0	0.91	0.11	1.15
B4 [35]	21.9	0.12	21.5	1.02	0.10	1.17	20.4	1.07	0.10	1.24	22.0	0.99	0.10	1.15
B5 [35]	22.6	0.14	22.0	1.02	0.10	1.37	20.9	1.08	0.09	1.45	22.2	1.02	0.10	1.36
B6 [35]	25.0	0.16	20.9	1.19	0.09	1.73	20.1	1.24	0.09	1.80	21.7	1.15	0.10	1.67
B7 [35]	20.2	0.11	20.7	0.98	0.10	1.04	19.5	1.04	0.10	1.10	21.0	0.96	0.11	1.02
B8 [35]	21.8	0.13	20.8	1.05	0.10	1.25	19.7	1.10	0.10	1.32	21.3	1.02	0.10	1.22
B9 [35]	19.6	0.11	20.0	0.98	0.10	1.09	20.3	0.97	0.10	1.07	22.0	0.89	0.11	0.99
C4 [35]	11.9	0.13	11.6	1.02	0.12	1.15	11.0	1.08	0.11	1.21	11.8	1.01	0.12	1.12
C5 [35]	14.0	0.17	11.6	1.21	0.11	1.46	11.0	1.27	0.11	1.53	11.9	1.17	0.12	1.41
C6 [35]	13.9	0.17	11.4	1.21	0.11	1.58	10.9	1.27	0.10	1.65	12.0	1.16	0.11	1.51
G3 [35]	27.1	0.10	30.5	0.89	0.09	1.01	28.7	0.94	0.09	1.08	31.0	0.87	0.10	1.00
G4 [35]	28.7	0.12	31.0	0.93	0.09	1.25	29.4	0.98	0.09	1.32	31.6	0.91	0.10	1.23
G5 [35]	29.5	0.11	29.8	0.99	0.09	1.27	28.5	1.03	0.09	1.33	30.7	0.96	0.09	1.24
G7 [35]	33.6	0.13	32.8	1.02	0.10	1.40	30.9	1.09	0.09	1.49	33.3	1.01	0.10	1.38
G8 [35]	33.6	0.12	31.2	1.08	0.09	1.32	29.3	1.15	0.09	1.40	31.5	1.07	0.09	1.30
I3 [35]	25.5	0.11	26.3	0.97	0.11	0.94	25.1	1.02	0.11	0.99	27.2	0.94	0.12	0.91
I4 [35]	28.0	0.12	26.5	1.06	0.11	1.11	25.2	1.11	0.11	1.17	27.3	1.03	0.11	1.08
I5 [35]	28.1	0.15	26.8	1.05	0.11	1.39	25.4	1.11	0.10	1.46	27.4	1.02	0.11	1.36
I6 [35]	27.5	0.13	26.9	1.02	0.11	1.19	25.7	1.07	0.10	1.24	27.7	0.99	0.11	1.15
J1 [35]	14.0	0.09	15.2	0.92	0.09	0.92	14.8	0.95	0.09	0.95	15.9	0.88	0.10	0.88
J2 [35]	17.1	0.12	15.4	1.10	0.09	1.35	14.3	1.19	0.09	1.45	15.7	1.09	0.09	1.32
J3 [35]	16.9	0.10	16.5	1.03	0.09	1.04	15.4	1.10	0.09	1.11	16.6	1.02	0.09	1.03
J4 [35]	18.0	0.11	15.8	1.13	0.09	1.26	15.1	1.19	0.08	1.33	16.3	1.10	0.09	1.23
K2 [35]	12.2	0.18	12.7	0.96	0.14	1.24	11.8	1.04	0.13	1.33	12.7	0.96	0.14	1.24
K3 [35]	12.4	0.19	12.3	1.01	0.14	1.40	11.0	1.13	0.12	1.56	12.2	1.02	0.14	1.42
K4 [35]	13.1	0.21	12.1	1.08	0.13	1.56	10.8	1.21	0.12	1.75	12.0	1.09	0.13	1.58
M1 [35]	19.2	0.11	19.7	0.97	0.10	1.11	20.7	0.93	0.10	1.05	22.5	0.85	0.11	0.97
M2 [35]	20.6	0.11	21.5	0.96	0.10	1.08	20.9	0.98	0.10	1.10	22.5	0.92	0.11	1.03
M3 [35]	20.7	0.12	20.7	1.00	0.10	1.19	19.5	1.06	0.10	1.27	21.0	0.98	0.10	1.18
M4 [35]	20.7	0.13	20.1	1.03	0.10	1.39	19.3	1.07	0.09	1.45	20.8	0.99	0.10	1.34
M5 [35]	21.7	0.12	20.6	1.05	0.10	1.27	19.6	1.11	0.09	1.34	21.3	1.02	0.10	1.23
M6 [35]	22.7	0.15	21.0	1.08	0.09	1.54	20.0	1.13	0.09	1.62	21.7	1.05	0.10	1.49
N1 [35]	7.6	0.13	6.9	1.10	0.16	0.79	6.5	1.17	0.15	0.83	7.0	1.08	0.17	0.77
N1a [35]	7.0	0.11	6.4	1.11	0.15	0.72	6.4	1.10	0.15	0.71	6.9	1.01	0.17	0.66
N2 [35]	7.4	0.22	7.0	1.06	0.16	1.36	6.5	1.14	0.15	1.45	7.1	1.05	0.16	1.34
N2a [35]	7.5	0.21	6.7	1.12	0.16	1.35	6.4	1.17	0.15	1.41	6.9	1.09	0.16	1.31
N3 [35]	7.4	0.21	6.7	1.11	0.16	1.34	6.2	1.19	0.15	1.43	6.7	1.10	0.16	1.33
N4 [35]	7.6	0.21	6.6	1.16	0.15	1.39	6.2	1.22	0.14	1.46	6.7	1.13	0.16	1.36
A2 [36]	11.3	0.12	13.6	0.83	0.13	0.92	13.1	0.86	0.13	0.96	14.0	0.81	0.13	0.89
A3 [36]	12.2	0.12	13.9	0.87	0.13	0.95	13.2	0.92	0.12	1.00	14.3	0.85	0.13	0.92
A4 [36]	12.5	0.15	14.0	0.90	0.13	1.16	13.2	0.95	0.12	1.22	14.3	0.88	0.13	1.14
B3 [36]	8.8	0.15	12.2	0.72	0.14	1.05	11.6	0.76	0.13	1.10	12.6	0.70	0.14	1.01
B4 [36]	10.2	0.15	12.3	0.83	0.14	1.07	11.7	0.87	0.13	1.12	12.6	0.81	0.14	1.04
B5UR1 [37]	11.6	0.09	12.4	0.93	0.14	0.63	12.0	0.97	0.13	0.65	13.1	0.89	0.14	0.60
B9UR1 [37]	13.0	0.13	16.4	0.79	0.15	0.90	16.0	0.81	0.14	0.92	17.3	0.75	0.16	0.86
B12UR1 [37]	16.2	0.09	16.9	0.96	0.15	0.61	16.5	0.98	0.15	0.62	17.8	0.91	0.16	0.57
B14UR1 [37]	19.3	0.12	17.9	1.08	0.15	0.77	17.4	1.11	0.15	0.79	18.9	1.02	0.16	0.73
B12UR2 [37]	17.8	0.11	16.6	1.07	0.15	0.74	16.1	1.10	0.15	0.76	17.4	1.02	0.16	0.71
B12UR3 [37]	16.0	0.10	15.9	1.01	0.14	0.71	15.8	1.01	0.14	0.71	17.3	0.93	0.16	0.65
B12UR4 [37]	16.9	0.14	16.1	1.05	0.14	0.97	16.1	1.05	0.14	0.97	17.5	0.96	0.16	0.89
B12UR5 [37]	13.6	0.04	16.8	0.81	0.15	0.24	16.4	0.83	0.14	0.25	17.8	0.76	0.16	0.23
H-06-12 [38]	75.0	0.09	65.2	1.15	0.07	1.19	76.5	0.98	0.08	1.02	83.5	0.90	0.09	0.93
H-07-10 [38]	70.5	0.09	67.2	1.05	0.08	1.18	72.8	0.97	0.08	1.09	79.0	0.89	0.09	1.01
H-07-16 [38]	65.3	0.09	69.8	0.93	0.08	1.08	73.0	0.89	0.08	1.03	79.2	0.82	0.09	0.95
H-12-12 [38]	77.1	0.07	78.9	0.98	0.09	0.86	78.6	0.98	0.09	0.87	85.0	0.91	0.09	0.80
H-12-16 [38]	79.3	0.09	79.2	1.00	0.09	1.07	78.6	1.01	0.09	1.08	85.3	0.93	0.09	0.99
H-14-10 [38]	61.8	0.09	76.9	0.80	0.09	0.98	74.7	0.83	0.09	1.01	80.6	0.77	0.09	0.94
H-20-20 [38]	76.0	0.09	83.5	0.91	0.09	1.03	80.6	0.94	0.09	1.07	87.1	0.87	0.09	0.99
N-06-06 [38]	43.2	0.08	53.3	0.81	0.07	1.07	54.7	0.79	0.08	1.04	59.3	0.73	0.08	0.96
N-06-12 [38]	51.8	0.11	54.5	0.95	0.08	1.51	54.7	0.95	0.08	1.51	59.1	0.88	0.08	1.39
N-07-10 [38]	41.6	0.11	55.2	0.75	0.08	1.39	53.5	0.78	0.08	1.44	57.6	0.72	0.08	1.33
N-07-16 [38]	40.0	0.11	52.5	0.76	0.07	1.49	53.4	0.75	0.07	1.46	57.5	0.70	0.08	1.36
N-12-12 [38]	49.3	0.09	57.5	0.86	0.08	1.16	55.4	0.89	0.08	1.21	59.2	0.83	0.08	1.13
N-12-16 [38]	57.1	0.12	57.6	0.99	0.08	1.54	55.0	1.04	0.07	1.61	59.2	0.96	0.08	1.50
N-14-10 [38]	41.8	0.12	56.8	0.74	0.08	1.52	53.7	0.78	0.08	1.60	57.9	0.72	0.08	1.49
N-20-20 [38]	55.0	0.13	58.5	0.94	0.08	1.62	54.6	1.01	0.07	1.74	58.9	0.93	0.08	1.61
SW12-1 [39]	32.3	0.15	36.1	0.89	0.13	1.14	33.8	0.96	0.13	1.20	35.7	0.91	0.16	0.98
SW10-1 [39]	24.6	0.13	24.5	1.00	0.14	0.95	22.7	1.08	0.13	1.03	24.7	1.00	0.19	0.70
SW10-2 [39]	29.6	0.20	30.1	0.98	0.14	1.47	28.7	1.03	0.13	1.49	31.5	0.94	0.15	1.33
SW10-3 [39]	26.6	0.15	25.1	1.06	0.14	1.12	23.5	1.13	0.13	1.18	27.6	0.96	0.15	1.01
SW10-4 [39]	27.7	0.16	27.0	1.02	0.14	1.19	25.2	1.10	0.13	1.26	29.4	0.94	0.15	1.08
SW8-1 [39]	19.7	0.16	19.2	1.03	0.14	1.14	18.5	1.07	0.14	1.13	19.4	1.02	0.19	0.84
SW8-2 [39]	22.5	0.14	19.8	1.14	0.14	1.02	18.7	1.20	0.14	1.06	21.7	1.04	0.16	0.92
D3 [35]	15.2	0.08	7.9	1.92	0.05	1.55	7.9	1.92	0.05	1.50	9.3	1.62	0.06	1.27
D4 [35]	15.8	0.12	8.7	1.83	0.06	2.14	8.7	1.82	0.06	2.10	10.2	1.55	0.07	1.79
T0 [40]	49.8	0.06	38.1	1.31	0.03	2.09	37.9	1.32	0.03	1.99	43.2	1.15	0.03	1.79
T1 [40]	48.0	0.04	33.0	1.45	0.03	1.46	32.8	1.46	0.03	1.39	37.9	1.27	0.04	1.22
T2 [40]	52.8	0.10	33.0	1.60	0.03	3.32	32.8	1.61	0.03	3.16	37.9	1.39	0.04	2.78
T5 [40]	62.5	0.06	86.3	0.72	0.07	0.91	49.9	1.25	0.03	2.08	56.5	1.11	0.03	1.92
VH1 [41]	12.0	0.12	9.7	1.24	0.07	1.66	9.7	1.24	0.07	1.66	9.9	1.21	0.10	1.23
VH2 [41]	11.5	0.07	10.2	1.12	0.08	0.90	10.3	1.12	0.08	0.91	11.0	1.05	0.10	0.73
A2 [42]	109.5	0.06	112.0	0.98	0.06	1.13	66.9	1.64	0.03	2.54	76.6	1.43	0.03	2.27
A3 [42]	113.3	0.06	128.4	0.88	0.04	1.37	67.5	1.68	0.03	2.27	77.7	1.46	0.03	1.98
A4 [42]	120.9	0.06	75.2	1.61	0.02	2.60	74.5	1.62	0.03	2.44	86.2	1.40	0.03	2.14
A5 [42]	120.9	0.04	74.7	1.62	0.02	1.82	74.1	1.63	0.03	1.72	85.6	1.41	0.03	1.50
B2 [42]	116.7	0.04	131.8	0.89	0.04	1.25	84.5	1.38	0.03	1.70	97.5	1.20	0.03	1.49
B3 [42]	130.5	0.05	134.1	0.97	0.03	1.31	92.3	1.41	0.03	1.76	105.3	1.24	0.03	1.57
B4 [42]	142.9	0.07	99.0	1.44	0.03	2.76	96.4	1.48	0.03	2.76	109.9	1.30	0.03	2.46
B5 [42]	146.3	0.06	97.8	1.50	0.03	2.54	95.2	1.54	0.03	2.54	109.4	1.34	0.03	2.21
C2 [42]	124.5	0.05	126.5	0.98	0.03	1.49	97.4	1.28	0.03	1.83	96.3	1.29	0.04	1.38
C3 [42]	131.9	0.06	129.7	1.02	0.03	1.94	98.1	1.34	0.03	2.47	112.4	1.17	0.03	2.18
C4 [42]	132.6	0.05	102.1	1.30	0.03	2.02	100.1	1.32	0.03	1.95	116.0	1.14	0.03	1.69
C5 [42]	138.3	0.05	106.4	1.30	0.02	2.06	104.2	1.33	0.03	2.00	119.5	1.16	0.03	1.75
C6 [42]	139.1	0.05	103.4	1.34	0.03	2.10	101.7	1.37	0.03	2.04	117.0	1.19	0.03	1.78
A095c [26]	102.9	0.03	101.6	1.01	0.04	0.80	99.2	1.04	0.04	0.83	102.9	1.00	0.05	0.64
A120a [26]	89.8	0.05	86.8	1.03	0.04	1.15	85.7	1.05	0.04	1.18	86.4	1.04	0.04	1.11
B065b [26]	54.4	0.03	58.0	0.94	0.03	1.33	58.0	0.94	0.03	1.24	67.4	0.81	0.03	1.09
B080a [26]	65.2	0.03	70.3	0.93	0.02	1.32	69.9	0.93	0.03	1.26	79.7	0.82	0.03	1.13
B110a [26]	128.3	0.04	128.5	1.00	0.04	0.96	125.9	1.02	0.04	1.01	127.1	1.01	0.05	0.80
C065a [26]	91.7	0.03	128.2	0.72	0.03	0.83	89.0	1.03	0.03	1.07	101.0	0.91	0.03	0.99
C100a [26]	122.2	0.03	134.3	0.91	0.03	0.85	128.6	0.95	0.03	0.87	134.8	0.91	0.04	0.67
D075a [26]	90.1	0.03	121.9	0.74	0.03	0.84	95.3	0.95	0.03	1.00	109.8	0.82	0.03	0.90
D090a [26]	96.1	0.03	110.3	0.87	0.03	1.14	107.8	0.89	0.03	1.10	122.8	0.78	0.03	0.98

**Table A4:** Cracking torques and corresponding twists (smeared constitutive laws *l*7 to *l*8).

Beam	$M_{Tcr}^{exp}$ kNm	$\theta_{\mathrm{cr}}^{\exp}$ °/m	$M_{Tcr}^{thl7}$ kNm	$\frac{M_{Tcr}^{exp}}{M_{Tcr}^{thl7}}$	$\theta_{\mathrm{cr}}^{\mathrm{thl}7}$ °/m	$\frac{\theta_{\mathrm{cr}}^{\exp}}{\theta_{\mathrm{cr}}^{\mathrm{thl}7}}$	$M_{Tcr}^{thl8}$ kNm	$\frac{M_{Tcr}^{exp}}{M_{Tcr}^{thl8}}$	$\theta_{\mathrm{cr}}^{\mathrm{thl}8}$ kNm	$\frac{\theta_{\mathrm{cr}}^{\exp}}{\theta_{\mathrm{cr}}^{\mathrm{thl}8}}$
B3 [35]	20.1	0.12	21.5	0.94	0.11	1.18	24.6	0.82	0.12	1.03
B4 [35]	21.9	0.12	22.1	0.99	0.10	1.14	24.3	0.90	0.11	1.04
B5 [35]	22.6	0.14	22.8	0.99	0.10	1.33	24.7	0.91	0.11	1.23
B6 [35]	25.0	0.16	21.9	1.14	0.10	1.65	23.7	1.05	0.11	1.53
B7 [35]	20.2	0.11	20.2	1.00	0.10	1.06	23.9	0.84	0.12	0.90
B8 [35]	21.8	0.13	20.6	1.06	0.10	1.26	24.2	0.90	0.12	1.07
B9 [35]	19.6	0.11	21.9	0.90	0.11	0.99	24.7	0.79	0.12	0.88
C4 [35]	11.9	0.13	11.9	0.99	0.12	1.11	13.1	0.90	0.13	1.01
C5 [35]	14.0	0.17	11.8	1.18	0.12	1.42	12.8	1.10	0.13	1.32
C6 [35]	13.9	0.17	11.8	1.18	0.11	1.54	12.8	1.09	0.12	1.42
G3 [35]	27.1	0.10	30.5	0.89	0.09	1.02	35.0	0.77	0.11	0.88
G4 [35]	28.7	0.12	31.6	0.91	0.10	1.23	35.5	0.81	0.11	1.09
G5 [35]	29.5	0.11	30.9	0.95	0.09	1.23	34.2	0.86	0.10	1.11
G7 [35]	33.6	0.13	33.1	1.01	0.10	1.38	37.5	0.90	0.11	1.22
G8 [35]	33.6	0.12	32.1	1.05	0.10	1.28	35.3	0.95	0.11	1.16
I3 [35]	25.5	0.11	27.0	0.94	0.12	0.92	30.4	0.84	0.13	0.81
I4 [35]	28.0	0.12	27.4	1.02	0.11	1.08	30.3	0.92	0.13	0.97
I5 [35]	28.1	0.15	27.8	1.01	0.11	1.34	30.2	0.93	0.12	1.23
I6 [35]	27.5	0.13	28.3	0.97	0.11	1.13	29.9	0.92	0.12	1.07
J1 [35]	14.0	0.09	15.3	0.92	0.09	0.92	18.1	0.77	0.11	0.77
J2 [35]	17.1	0.12	15.3	1.11	0.09	1.36	17.6	0.97	0.11	1.18
J3 [35]	16.9	0.10	16.5	1.03	0.09	1.04	18.6	0.91	0.10	0.92
J4 [35]	18.0	0.11	16.4	1.09	0.09	1.22	18.1	0.99	0.10	1.10
K2 [35]	12.2	0.18	12.8	0.95	0.15	1.22	14.0	0.87	0.16	1.12
K3 [35]	12.4	0.19	12.4	1.01	0.14	1.40	13.1	0.95	0.15	1.32
K4 [35]	13.1	0.21	12.2	1.07	0.13	1.55	12.5	1.05	0.14	1.51
M1 [35]	19.2	0.11	21.9	0.88	0.11	0.99	25.4	0.76	0.12	0.86
M2 [35]	20.6	0.11	22.4	0.92	0.11	1.03	25.3	0.81	0.12	0.92
M3 [35]	20.7	0.12	21.1	0.98	0.10	1.17	23.4	0.88	0.11	1.06
M4 [35]	20.7	0.13	21.2	0.98	0.10	1.32	22.9	0.90	0.11	1.22
M5 [35]	21.7	0.12	22.0	0.99	0.10	1.19	23.1	0.94	0.11	1.13
M6 [35]	22.7	0.15	22.6	1.01	0.10	1.43	23.4	0.97	0.11	1.38
N1 [35]	7.6	0.13	7.1	1.07	0.17	0.77	8.0	0.95	0.19	0.68
N1a [35]	7.0	0.11	6.9	1.01	0.16	0.66	7.9	0.89	0.19	0.58
N2 [35]	7.4	0.22	7.3	1.02	0.17	1.30	8.0	0.93	0.18	1.19
N2a [35]	7.5	0.21	7.0	1.08	0.16	1.29	7.6	0.98	0.18	1.18
N3 [35]	7.4	0.21	7.0	1.06	0.17	1.28	7.6	0.97	0.18	1.18
N4 [35]	7.6	0.21	7.0	1.08	0.16	1.30	7.5	1.01	0.17	1.22
A2 [36]	11.3	0.12	13.8	0.82	0.13	0.91	15.9	0.71	0.15	0.79
A3 [36]	12.2	0.12	14.3	0.86	0.13	0.93	16.0	0.76	0.15	0.83
A4 [36]	12.5	0.15	14.4	0.87	0.13	1.13	15.8	0.79	0.15	1.03
B3 [36]	8.8	0.15	13.0	0.68	0.15	0.99	14.2	0.62	0.16	0.90
B4 [36]	10.2	0.15	13.0	0.78	0.15	1.01	14.0	0.73	0.16	0.93
B5UR1 [37]	11.6	0.09	13.0	0.89	0.14	0.60	14.8	0.79	0.16	0.53
B9UR1 [37]	13.0	0.13	17.2	0.76	0.16	0.86	19.7	0.66	0.18	0.75
B12UR1 [37]	16.2	0.09	17.8	0.91	0.16	0.58	20.2	0.80	0.18	0.51
B14UR1 [37]	19.3	0.12	18.8	1.02	0.16	0.73	21.5	0.90	0.18	0.64
B12UR2 [37]	17.8	0.11	17.4	1.03	0.16	0.71	19.9	0.89	0.18	0.62
B12UR3 [37]	16.0	0.10	17.5	0.91	0.16	0.64	19.5	0.82	0.18	0.58
B12UR4 [37]	16.9	0.14	18.0	0.94	0.16	0.87	19.7	0.86	0.18	0.80
B12UR5 [37]	13.6	0.04	18.0	0.75	0.16	0.22	20.0	0.68	0.18	0.20
H-06-12 [38]	75.0	0.09	85.0	0.88	0.09	0.92	94.2	0.80	0.10	0.83
H-07-10 [38]	70.5	0.09	77.1	0.91	0.09	1.03	89.5	0.79	0.10	0.89
H-07-16 [38]	65.3	0.09	80.3	0.81	0.09	0.94	87.5	0.75	0.10	0.86
H-12-12 [38]	77.1	0.07	87.0	0.89	0.10	0.78	96.3	0.80	0.11	0.71
H-12-16 [38]	79.3	0.09	86.5	0.92	0.09	0.98	94.6	0.84	0.10	0.89
H-14-10 [38]	61.8	0.09	78.8	0.78	0.09	0.96	90.5	0.68	0.10	0.83
H-20-20 [38]	76.0	0.09	89.7	0.85	0.10	0.96	96.2	0.79	0.10	0.89
N-06-06 [38]	43.2	0.08	57.1	0.76	0.08	1.00	67.4	0.64	0.09	0.85
N-06-12 [38]	51.8	0.11	60.5	0.86	0.08	1.36	66.5	0.78	0.09	1.24
N-07-10 [38]	41.6	0.11	56.3	0.74	0.08	1.36	64.9	0.64	0.09	1.18
N-07-16 [38]	40.0	0.11	58.4	0.68	0.08	1.33	64.0	0.63	0.09	1.22
N-12-12 [38]	49.3	0.09	60.5	0.81	0.08	1.10	66.8	0.74	0.09	1.00
N-12-16 [38]	57.1	0.12	59.9	0.95	0.08	1.48	65.8	0.87	0.09	1.35
N-14-10 [38]	41.8	0.12	56.5	0.74	0.08	1.52	64.1	0.65	0.09	1.34
N-20-20 [38]	55.0	0.13	61.2	0.90	0.08	1.55	64.7	0.85	0.09	1.47
SW12-1 [39]	32.3	0.15	38.7	0.84	0.15	1.04	41.3	0.78	0.16	0.97
SW10-1 [39]	24.6	0.13	26.1	0.94	0.15	0.87	27.8	0.88	0.16	0.82
SW10-2 [39]	29.6	0.20	32.1	0.92	0.15	1.34	34.6	0.86	0.16	1.24
SW10-3 [39]	26.6	0.15	26.6	1.00	0.15	1.04	28.7	0.93	0.16	0.96
SW10-4 [39]	27.7	0.16	28.9	0.96	0.15	1.09	30.3	0.92	0.15	1.04
SW8-1 [39]	19.7	0.16	21.1	0.93	0.16	0.97	21.9	0.90	0.16	0.98
SW8-2 [39]	22.5	0.14	21.1	1.07	0.15	0.94	22.7	0.99	0.17	0.87
D3 [35]	15.2	0.08	8.4	1.81	0.06	1.42	9.1	1.67	0.06	1.32
D4 [35]	15.8	0.12	9.4	1.69	0.06	1.94	10.0	1.58	0.06	1.82
T0 [40]	49.8	0.06	41.2	1.21	0.03	1.78	44.1	1.13	0.04	1.68
T1 [40]	48.0	0.04	34.9	1.38	0.03	1.29	38.8	1.24	0.04	1.15
T2 [40]	52.8	0.10	34.9	1.51	0.03	2.93	38.8	1.36	0.04	2.63
T5 [40]	62.5	0.06	53.1	1.18	0.03	1.92	59.5	1.05	0.04	1.71
VH1 [41]	12.0	0.12	10.1	1.19	0.08	1.60	11.8	1.02	0.09	1.37
VH2 [41]	11.5	0.07	11.4	1.01	0.09	0.82	12.4	0.93	0.09	0.76
A2 [42]	109.5	0.06	67.7	1.62	0.03	2.49	79.5	1.38	0.03	2.09
A3 [42]	113.3	0.06	69.1	1.64	0.03	2.17	80.0	1.42	0.03	1.87
A4 [42]	120.9	0.06	77.9	1.55	0.03	2.34	87.7	1.38	0.03	2.05
A5 [42]	120.9	0.04	78.4	1.54	0.03	1.61	87.0	1.39	0.03	1.44
B2 [42]	116.7	0.04	86.4	1.35	0.03	1.69	99.8	1.17	0.03	1.44
B3 [42]	130.5	0.05	96.6	1.35	0.03	1.66	108.8	1.20	0.03	1.47
B4 [42]	142.9	0.07	100.7	1.42	0.03	2.60	112.4	1.27	0.03	2.36
B5 [42]	146.3	0.06	100.0	1.46	0.03	2.42	110.6	1.32	0.03	2.17
C2 [42]	124.5	0.05	99.0	1.26	0.03	1.79	115.2	1.08	0.03	1.56
C3 [42]	131.9	0.06	102.9	1.28	0.03	2.33	116.0	1.14	0.03	2.04
C4 [42]	132.6	0.05	105.4	1.26	0.03	1.86	117.1	1.13	0.03	1.66
C5 [42]	138.3	0.05	109.5	1.26	0.03	1.88	122.1	1.13	0.03	1.67
C6 [42]	139.1	0.05	109.5	1.27	0.03	1.87	118.1	1.18	0.03	1.75
A095c [26]	102.9	0.03	100.2	1.03	0.04	0.82	120.2	0.86	0.05	0.68
A120a [26]	89.8	0.05	87.6	1.03	0.04	1.15	104.2	0.86	0.05	0.95
B065b [26]	54.4	0.03	62.0	0.88	0.03	1.16	66.8	0.81	0.03	1.07
B080a [26]	65.2	0.03	72.0	0.91	0.03	1.22	81.3	0.80	0.03	1.09
B110a [26]	128.3	0.04	128.6	1.00	0.04	0.99	152.2	0.84	0.05	0.82
C065a [26]	91.7	0.03	92.8	0.99	0.03	1.01	105.7	0.87	0.03	0.88
C100a [26]	122.2	0.03	133.1	0.92	0.03	0.84	153.1	0.80	0.04	0.72
D075a [26]	90.1	0.03	100.1	0.90	0.03	0.95	111.8	0.81	0.03	0.85
D090a [26]	96.1	0.03	110.6	0.87	0.03	1.09	126.0	0.76	0.03	0.94
